# Elagolix treatment in women with heavy menstrual bleeding associated with uterine fibroid: a systematic review and meta-analysis

**DOI:** 10.1186/s12905-022-01596-2

**Published:** 2022-01-15

**Authors:** Juliawati Muhammad, Yusnita Yusof, Imran Ahmad, Mohd Noor Norhayati

**Affiliations:** Department of Family Medicine, School of Medical Sciences, Universiti Sains Malaysia, 16150 Kubang Kerian, Kelantan Malaysia

**Keywords:** Elagolix, GnRH antagonist, Uterine fibroid, Leiomyoma, Heavy menstrual bleeding, Estradiol/norethindrone acetate

## Abstract

**Background:**

Elagolix is effective and safe for treating menorrhagia in women with uterine fibroid. However, it is reported to be associated with hypoestrogenism that can be alleviated by adding estradiol/norethindrone acetate. This systematic review and meta-analysis aimed to determine the effectiveness of elagolix treatment in women with heavy menstrual bleeding associated with uterine fibroid by comparing: elagolix versus placebo and elagolix versus estradiol/norethindrone acetate.

**Methodology:**

The Cochrane Central Register of Controlled Trials (CENTRAL 2021, Issue 3 of 12), MEDLINE databases (1980 to December week 1, 2020), and trial registries for relevant randomized clinical trials were used. All randomized clinical trials were reviewed and evaluated. Random effects models were used to estimate the dichotomous outcomes and mean differences with 95% confidence intervals. Data for risk of bias, heterogeneity, sensitivity, reporting bias and quality of evidence were assessed.

**Results:**

Four randomized controlled trials with 1949 premenopausal women from 323 locations were included. Elagolix improved menstrual blood loss of less than 80 ml (RR 4.81, 95% CI 2.45 to 9.45; four trials, 869 participants; moderate quality evidence) or more than 50% reduction from baseline (RR 4.87, 95% CI 2.55 to 9.31; four trials, 869 participants; moderate quality evidence) compared to placebo. There was no difference in menstrual blood loss of less than 80 ml (RR 1.08, 95% CI 1.00 to 1.16; five trials, 1365 participants; moderate quality evidence) or more than 50% reduction from baseline between the elagolix (RR 1.08, 95% CI 1.01 to 1.15; five trials, 1365 participants; high quality evidence) and elagolix with estradiol/norethindrone acetate. In both comparisons, elagolix has reduced the mean percentage change in uterine and fibroid volume, improved symptoms, and health-related quality of life. More patients had hot flush, and bone mineral density loss in the elagolix treatment compared to both placebo and elagolix with estradiol/norethindrone acetate.

**Conclusions:**

Elagolix appeared to be effective in reducing heavy menstrual bleeding caused by uterine fibroid and combination with estradiol/norethindrone acetate was able to alleviate the hypoestrogenism side effects in premenopausal women.

*Review registration*

PROSPERO CDR 42021233898.

**Supplementary Information:**

The online version contains supplementary material available at 10.1186/s12905-022-01596-2.

## Background

Uterine fibroids are benign and noncancerous monoclonal tumor arising from smooth muscle cells and fibroblasts of the myometrium. It is believed that the specific growth factor receptor, hyper-estrogenic effect, angiogenesis disorder, and altered smooth muscle cell proliferation have a vital role in uterine fibroid growth [1]. Most women are asymptomatic. If symptomatic, they may present with abnormal bleeding (e.g., heavy bleeding, prolonged bleeding or irregular periods), pelvic pain, and dyspareunia [2]. The fibroids may also compromise reproductive functions, possibly contributing to subfertility, pregnancy outcomes, health related quality of life, economic burden, and work productivity [3–5].

The estimated prevalence of uterine fibroids increases with age and varies from 5.4 to 23.6% during the reproductive years [6]. The approximate prevalence of uterine fibroid was 33% based on clinical assessment, 50% with an ultrasound scan and 77% with histological examination of hysterectomy specimens [7]. Treatments can be nonhormonal, hormonal pharmacological compounds, and surgical treatment. The surgical options include myomectomy, hysterectomy, endometrial uterine artery embolization, and endometrial ablation [4, 8, 9].

Elagolix is a newly synthesized nonpeptidic gonadotrophin-releasing hormone (GnRH) receptor blocker that has been developed to treat endometriosis. It has recently received US FDA approval in July 2018 to manage moderate to severe pain associated with endometriosis [10]. The elagolix also has given positive feedback or benefit in treating women with uterine fibroids by reducing pain and heavy menses. GnRH antagonist is a synthetic peptide structurally analogous to the natural GnRH hormones that bind to GnRH receptors causing gonadotropin suppression [11–13]. The circulating estrogen and progesterone level will be suppressed by shutting down the pituitary-ovarian axis. The suppression in steroid hormone level will cause the fibroid to shrink, reduce a significant menstrual blood loss, uterine volume, fibroid volume and achieve amenorrhea, which will later improve the hemoglobin level [8]. It also reduces symptom severity and improves the health-related quality of life.

Other medical interventions include nonhormonal, hormonal medication, and surgical approaches that have been approved as beneficial in fibroid management. However, a certain treatment has limited usage due to substantial adverse effects of hypoestrogenism, e.g., hot flush, reduces bone mineral density, which is a risk factor of osteoporosis later on. Elagolix has a better adverse events profile. The addition of estradiol/norethindrone acetate to the treatment regime can prevent bone loss due to hypoestrogenic effect, which increases the safety of overall elagolix treatment [14].

This systematic review and meta-analysis aimed to determine the effectiveness of elagolix treatment in women with heavy menstrual bleeding associated with a uterine fibroid. Even though it is known to have a better tolerable safety profile, side effects of hypoestrogenism are commonly reported and can be relieved with the addition of estradiol/norethindrone acetate. The evaluation incorporated two comparisons: elagolix versus placebo; elagolix versus estradiol/norethindrone acetate. This will give a beneficial outcome for the patients and eventually improve their quality of life. Elagolix may be a part of the clinical application as one option for treating symptomatic uterine fibroid effectively and reducing undesirable side effects. Different dosages of elagolix were evaluated to determine its efficacy in reducing heavy and prolonged menstrual blood loss associated with uterine fibroids.

## Materials and methods

We conducted this systematic review according to the protocol previously published in the PROSPERO register (https://www.crd.york.ac.uk/PROSPERO), [CDR42021233898]. The types of studies included were randomized control trials (RCTs) comparing elagolix with placebo or estradiol/norethindrone acetate. We included double-blinded studies.

### Eligibility criteria

We included nonpregnant, premenopausal women aged 18–51 years old who had severe menstrual bleeding, identified as more than 80 ml of menstrual blood loss per menstrual cycle for at least two separate cycles as assessed by the validated alkaline hematin method. They should have documented uterine fibroids confirmed by either transabdominal or transvaginal ultrasound. The type of intervention was elagolix compared to placebo or estradiol/norethindrone acetate. The primary outcome was the number of participants having a reduction of menstrual blood loss of less than 80 ml or more than 50% in menstrual blood loss. Secondary outcomes were looking at improvement in hemoglobin level, uterine and fibroid volume, symptoms severity, health-related quality of life, bone mineral density and adverse events.

Heavy menstrual bleeding was defined as blood loss of or exceeding 80 ml per menstrual cycle and measured by the standard validated alkaline hematin method [15–17]. A 50% cut-off point was chosen because blood comprised 50% of total menstrual flow in women with excessive menstrual blood loss of more than 100 ml [18]. The follow-up period for the primary outcome was at least twelve weeks after intervention. The primary outcome was measured during the last month of the treatment period.

### Search strategies

Since 1980 was the year that gonadotropin-releasing hormone analogs were first adopted for medical use, we searched the Cochrane Central Register of Controlled Trials (CENTRAL 2021, Issue 3 of 12) and MEDLINE databases (1980 to December week 1, 2020). The keywords applied were (menorrhagia OR heavy menstrual bleeding OR abnormal uterine bleeding OR excessive menstrual bleeding) AND (fibroid OR leiomyoma OR fibroma OR fibromyoma) AND (elagolix OR GnRH antagonist). We used the search strategy in Additional file 1 to search MEDLINE, CENTRAL, and other databases. We restricted the publications to the English language only. We checked the reference list of identified randomized controlled trials and reviewed articles to find unpublished trials or trials not identified by electronic searches. We also contacted experts in the field and pharmaceutical companies that market elagolix to identify unpublished trials. We searched for ongoing trials through the World Health Organization International Clinical Trials Registry Platform https://www.who.int/ictrp/en and www.clinicaltrials.gov.

### Trial selection

We scanned the titles and abstracts from the searches. We obtained full-text articles when they appear to meet the eligibility criteria, or insufficient information to assess the eligibility. We assessed the eligibility of the trials independently and documented the reasons for exclusion. We resolved any disagreements between the review authors by discussion. We contacted the authors if clarification was needed. We excluded papers in languages other than English.

### Data extraction

We extracted data from each of the selected trials by using data extraction forms which include study setting, participant characteristics (age, sex, ethnicity), methodology (number of participants randomized and analyzed, duration of follow-up), dosage of elagolix, dosage of estradiol/norethindrone acetate, reduction of menstrual blood loss of less than 80 ml, reduction of more than 50% menstrual blood loss, uterine volume, fibroid volume, symptoms severity, health-related quality of life, haemoglobin level, bone mineral density, and adverse event medication (Additional file 3).

### Risk of bias assessment

We assessed the risk of bias based on random sequence generation, allocation concealment, blinding of participants and personnel, blinding of outcome assessors, completeness of outcome data, the selectivity of outcome reporting and other bias [19]. We resolved any disagreements by discussion. If there were sufficient studies, we intended to use funnel plots to assess the possibility of reporting biases or small study biases, or both.

### Statistical analysis

We planned to undertake meta-analyses using Review Manager 5.4 software [20] and examined heterogeneity using a random-effects model to pool data. We measured the treatment effect for dichotomous outcomes using risk ratios and absolute risk reduction, and for continuous outcomes we used mean differences; both with 95% confidence intervals. We performed a sensitivity analysis to investigate the risk of bias for sequence generation and allocation concealment of included studies. We contacted the original trial authors to request missing or inadequately reported data. We performed analyses on the available data in the event that missing data was not available.

The planned subgroup analyses were dosage of elagolix and dosage of estradiol/norethindrone acetate. We were unable to carry out the subgroup dosage of elagolix as outlined in the protocol because there were insufficient trials. However, we conducted subgroup analyses on the frequency of drug administration either twice daily (bd) or once daily (qd) administration, uterine volume and fibroid volume.

### Assessment of heterogeneity

We assessed the presence of heterogeneity in two steps. First, we assessed obvious heterogeneity at face value by comparing populations, settings, interventions, and outcomes. Second, we assessed statistical heterogeneity by means of the I^2^ statistic [19]. The threshold for the interpretation of the I^2^ statistic can be misleading, since the importance of inconsistency depends on several factors. We planned to use the guide to interpretation of heterogeneity as outlined: 0% to 40% might not be important; 30% to 60% may represent moderate heterogeneity; 50% to 90% may represent substantial heterogeneity; and 75% to 100% would be considerable heterogeneity [19].

### Grading quality of evidence

We assessed the quality of evidence for primary and secondary outcomes according to GRADE methodology [21] for risk of bias, inconsistency, indirectness, imprecision, and publication bias; classified as very low, low, moderate, or high. Quality can be downgraded depending on the presence of four factors: (i) limitations in the design and implementation of available studies; (ii) indirectness of evidence; (iii) unexplained heterogeneity or inconsistency of results; and (iv) imprecision of results.

## Results

### Results of the search

**Fig. 1:**
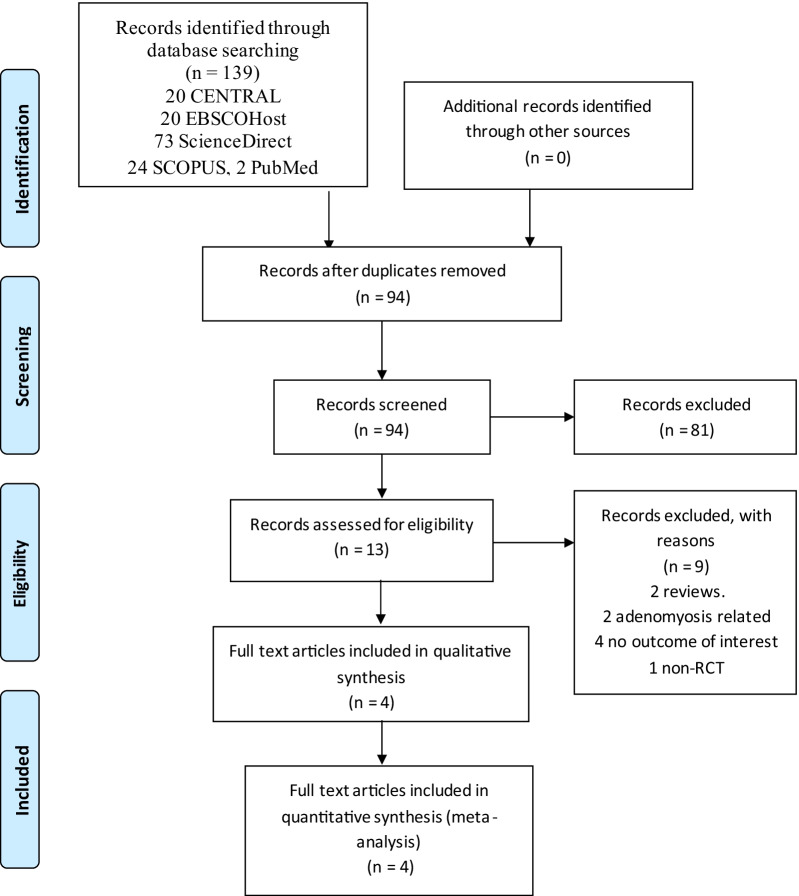
PRISMA flow diagram

We retrieved 139 records from the search of the electronic database and no other records from other sources (Fig. 1). A total of 94 records were screened after duplicates were removed. We reviewed full copies of 13 and assessed them for eligibility. We identified four articles as possibly meeting the review inclusion criteria, and nine of them were ineligible for inclusion. One article was a non-randomized controlled trial that evaluated the clinical response of elagolix-treated women who did not achieve the primary outcome [22]. Two reviews, one on predictors of response to elagolix with add-back therapy and the other on medical treatment of uterine leiomyoma, were relevant to our research query [4, 23]. There was no outcome of interest in the four papers as two papers [3, 11] on elagolix pharmacotherapy and pharmacodynamics and another two more papers [14, 24] on drug-drug interactions were written. Adenomyosis was the topic of two more publications [25, 26]. We attempted to contact the trial authors for the full article but received no response. Therefore, we included four trials.

### Included studies

Four randomized controlled trials with 1949 participants were included in the study [27–30]. All four trials reported the primary outcome. All trials were sponsored by AbbVie [27–30].

### Participants

All four studies were carried out in 323 locations across the United States, Puerto Rico, and Canada. One trial recruited participants from clinic settings [27]. The other three trials did not mention the location from which participants were recruited [28–30]. Three studies included premenopausal women aged 18 to 51 at the screening time [28–30], while one study recruited participants aged 20–49 [27]. They underwent ultrasonography-confirmed diagnosis of uterine fibroids and heavy menstrual bleeding, as characterized by more than 80 ml of menstrual blood loss per menstrual cycle for at least two cycles. The trials excluded participants due to a complex ovarian cyst, cancer, pelvic inflammatory disorder, osteoporosis history, or metabolic bone disease. Participants who had a myomectomy or hysterectomy for symptomatic uterine fibroid were exempted from the study [27–30].

### Intervention

Participants in the trials were randomized to the intervention and comparison groups. Two identical, double-blind, randomized, placebo-controlled, six-month phase 3 trials (Elaris Uterine Fibroids 1 and Elaris Uterine Fibroid 2) have been reported in one trial [29]. Elaris Uterine Fibroid-1 and Elaris Uterine Fibroid-2 participants were later randomized or pooled into a new study [30] to look at the long-term twelve-month safety and efficacy of elagolix with or without estradiol/norethindrone acetate. The meta-analysis included four trials that evaluated the primary outcomes. Three trials compared elagolix with placebo [27–29], and four trials compared to elagolix with estradiol/norethindrone acetate [27–30]. Only one trial compared elagolix to placebo at different doses of 100 mg bd, 200 mg bd, 300 mg bd, 400 mg qd, and 600 mg qd [27]. One study was compared to placebo at doses of 300 mg bd and 600 mg qd [28]. Another trial was compared elagolix to placebo at a dose of 300 mg bd [29].

In a comparison of elagolix to elagolix with estradiol/norethindrone acetate, one trial compared it at a dose of 0.5 mg estradiol/0.1 mg norethindrone acetate [27], while two trials compared it at a dose of 1.0 mg estradiol/0.5 mg norethindrone acetate [29, 30]. In one trial, elagolix was compared to elagolix with estradiol/norethindrone acetate at two doses: 0.5 mg estradiol/0.1 mg norethindrone acetate and 1.0 mg estradiol/0.5 mg norethindrone acetate [28]. All medications are taken orally as tablets or capsules. The duration of treatment differed between trials compared to elagolix versus placebo, as only one trial was three months [27], and the other two trials were six months [28, 29]. In contrast, the length of treatment differed between trials when comparing elagolix to elagolix with estradiol/norethindrone acetate, with a three-month [27], a six-month [28, 29], and a twelve-month [30] period.

### Outcomes

The validated alkaline hematin method was used to quantify and evaluate the primary outcome in all four trials [27–30]. Any spotting or bleeding episodes on a sanitary pad were reported at the time of screening or during treatment. Participants kept an electronic daily bleeding diary (eDiary) and assessed bleeding patterns using the validated Mansfield-Voda-Jorgenson Menstrual Bleeding Scale [31]. All studies were followed up to at least three-months duration. The primary outcome was measured during the last month of the treatment period.

All four trials reported all secondary outcomes except for one study [27], which did not record bone mineral density due to a limited study time and a small sample size per group. Reduction of uterine and fibroid volume was calculated using trans abdominal or transvaginal ultrasound. The mean percentage change from baseline to the end of the treatment month was recorded.

The Uterine Fibroid Symptom and Quality of Life questionnaire’s cumulative score were used to measure symptom severity reduction and change in health-related quality of life in women with symptomatic uterine fibroids. It was a disease-specific, self-administered, validated questionnaire. There were 37 questions in all, split into two parts. The first part consisted of an 8-item symptom severity scale. The second part consisted of a 29-item health-related quality of life subscale with six domains (concern, behaviors, energy/mood, power, self-consciousness, and sexual function). All items are rated on a 5-point scale, with symptom intensity items ranging from “not at all” to “a great deal”, and health-related quality of life items ranging from “none of the time” to “all of the time”. The cumulative score for each of the two components was determined by adding the symptom intensity and health-related quality of life subscale scores and translating them to a 0-to-100-point scale. Higher overall health-related quality of life scores indicated better quality of life, while lower symptom severity scores indicate better quality of life.

The percentage of increase in hemoglobin concentration from baseline to the last month of treatment was reported in all trials. Loss of bone mineral density was assessed by dual-energy x-ray absorptiometry scans of the lumbar spine, total hip, and femoral neck. The mean percentage change in bone mineral density from baseline to the last month of treatment was recorded in three studies [28–30]. Any adverse events were recorded beginning with the first dose of the study drug and continuing for up to 30 days after completing the last dose of the study drug. All four trials identified common adverse events such as hot flushes, headaches, nausea, and fatigue. In this review, only two trials documented adverse events such as abdominal pain, dizziness, and hypertension [27, 28]. Other non-significant adverse events identified in clinical trials will not be addressed in this review.

### Risk of bias in included studies

**Fig. 2:**
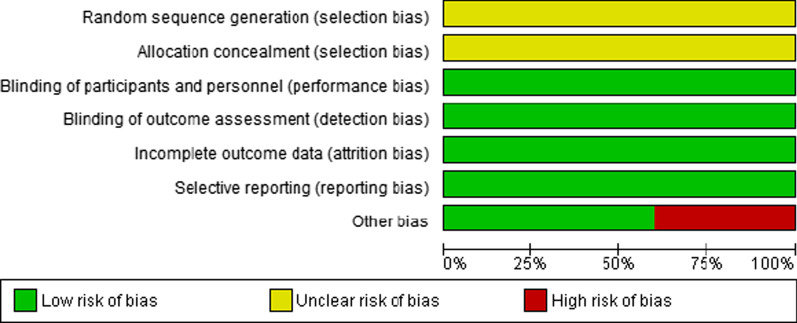
‘Risk of bias’ graph: review authors’ judgements about each risk of bias item presented as percentages across all included studies

**Fig. 3:**
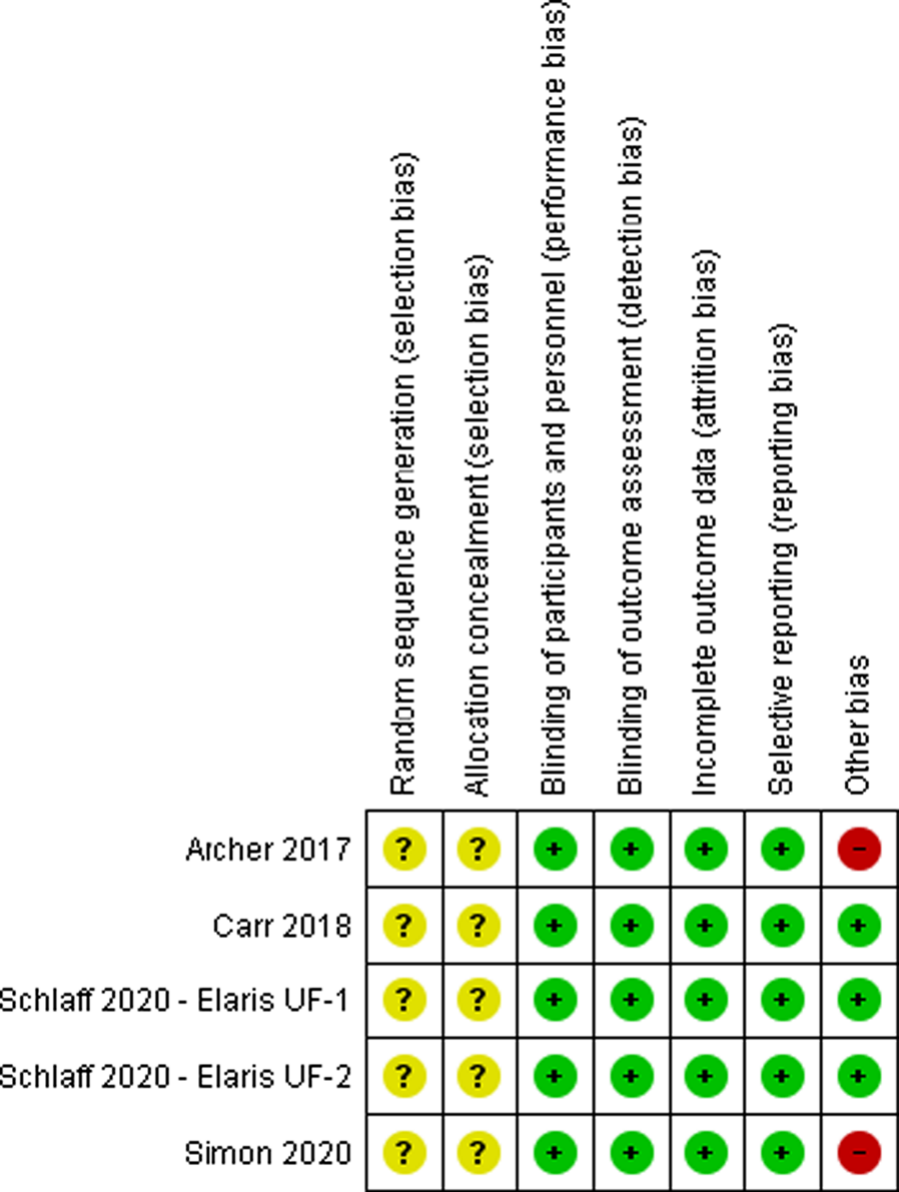
’Risk of bias’ summary: review authors’ judgements about each risk of bias item for each included study

The assessment risk of bias is shown in Figs. 2 and 3. Figure 2 shows the proportion of studies assessed as low, high or unclear risk of bias for each risk of bias indicator. Figure 3 shows the risk of bias indicators for individual studies. The details of these trials are found in the table of characteristics of included studies (Table 1).

**Table 1:** Characteristics of included trial

Reference	Country	Participants		Inclusion study period/ Treatment period	Intervention	Elagolix dosage and frequency administration	Comparator	No of participants/ No of trial sites	No of missing
		Age group; mean baseline MBL	Mean baseline uterine volume / mean baseline fibroid volume						
Archer 2017	USA	20–49 years; 267 mL	535 ± 389 cm^3^ / 91 ± 175 cm^3^	September 2011-May 2014/ 3 months	Elagolix (ABT-620)	i; 100 mg bd ii; 200 mg bd iii; 300 mg bd iv; 400 mg qd v; 600 mg qd	i; placebo (matching placebo tablet) ii; 0.5 mg E2/ 0.1 md NETA	271/ 45	Intervention (29) Control (14)
Carr 2018	USA	18–51 years; 246 ± 180 mL	628 ± 462 cm^3^/ 150 ± 196 cm^3^	April 2013-Dec 2015/ 6 months	Elagolix (ABT-620)	i; 300 mg bd ii; 600 mg qd	i; placebo (Oral coated placebo) ii; 0.5 mg E2/ 0.1 mg NETA iii; 1.0 mg E2/ 0.5 mg NETA	571*/ 86 *4 women were randomized but not treated	Intervention (32) Control (97)
Schlaff 2020	USA	(UF-1) 18–51 years; 245 ± 161 mL (UF-2) 18–51 years; 234 ± 156 mL	(UF-1) 482 ± 393 cm^3^ / 50 ± 68.9 cm^3^ (UF-2) 519 ± 437 cm^3^ / 63 ± 111 cm^3^	(UF-1) Dec 2015- Dec 2018/ 6 months (UF-2) Feb 2016 – Feb 2018 / 6 months	Elagolix (ABT-620)	i; 300 mg bd	i; placebo (film coated placebo tab) ii; 1.0 mg E2/ 0.5 mg NETA	(UF-1) 413 (UF-2) 378 /77	(UF-1) Intervention (23) Control (62) (UF-2) Intervention (26) Control (63)
Simon 2020 UF EXTEND	USA	18–51 years; 236 ± 159 mL	519 ± 457 cm^3^ / 59 ± 97 cm^3^	September 2016- Mac 2019/ 12 months	Elagolix (ABT-620)	i; 300 mg bd	i; 1.0 mg E2/ 0.5 mg NETA	316 out of 433 recruited*/ 115 *117 placebo participants exempted	Intervention (19) Control (36)

MBL-menstrual blood loss; UF-1—elaris uterine fibroid-1; UF-2—elaris uterine fibroid -2; RCT-randomized controlled trial; USA-united states of America; bd-twice daily; qd-once daily; E2—estradiol; NETA—norethindrone acetate

^*^117 placebo participants in pivotal study (Schlaff 2020) exempted as not fulfills eligibility criteria; UF-EXTEND-Uterine Fibroid extend is an additional 6-month for total up to 12-month treatment period

Add-back therapy; elagolix with estradiol/norethindrone acetate

### Allocation

Only one trial, with 271 participants, was reported to have been recruited in a clinic setting, while the other three were not [27]. The method of randomization was not reported in all four trials [27–30]. Thus, we judged random sequence generation as having an unclear risk of bias. Allocation concealment was not mentioned and regarded as unclear in four trials [27–30].

### Blinding

Participants, care provider, investigator and outcome assessor were masked in all four trials. The details on blinding were not recorded in all four trials, but the outcomes were unlikely to be influenced as it was objectively collected and measured using standardized methods [27–30]. Therefore, they are judged as having a low risk of bias.

### Incomplete outcome data

More than 80% of participants completed the studies in two trials [27, 30]. Meanwhile, 74.4% of participants in one trial completed the study [28]. Approximately 129 of the 571 participants failing to complete the analysis due to hypoestrogenism side effects (n = 39), withdrawal (n = 38), loss of follow up (n = 25), noncompliance (n = 11), lack of efficacy (n = 3), surgery (n = 4) and other (n = 9) [28]. About 78% of 791 participants completed studies in Elaris Uterine Fibroid-1 and Elaris Uterine Fibroid-2 [29]. The study drug was discontinued by similar proportions of women in both treatment groups (16.5% for elagolix with estradiol/norethindrone acetate and 19.4% for elagolix alone), with the most common primary reason being lost to follow-up (5.0% and 5.1%, respectively) in one trial [29]. Missing data were evenly balanced across groups, and the reasons were similar. The most common reasons for missing outcome data included withdrawal, noncompliance, loss to follow up, hypoestrogenism side effects, pregnancy, and surgery, which led to discontinuation.

### Selective reporting

All four trials reported the outcomes as specified in their methods section [27–30]. The outcomes listed in the registered protocol were those reported. Although changes in bone mineral density were assessed as an exploratory parameter, one trial did not report due to the short duration of the study and the relatively small sample size per group [27]. We graded it as having a low risk of bias.

### Other potential source of bias

We discovered that women with asymptomatic anemia and a hemoglobin level of less than 12 g/dl at screening or during the study period were advised to take iron supplements in two trials [27, 30]. This could have an influence on the hemoglobin level at the end of the treatment period. Thus, we judged it as having a high risk of bias. We detected no other potential source of bias in the other two trials [28, 29].

### Effects of intervention

There would be two comparisons evaluated in this review, i.e., comparing elagolix versus placebo and comparing elagolix versus estradiol/norethindrone acetate.

#### Comparison between elagolix and placebo

**Fig. 4:**
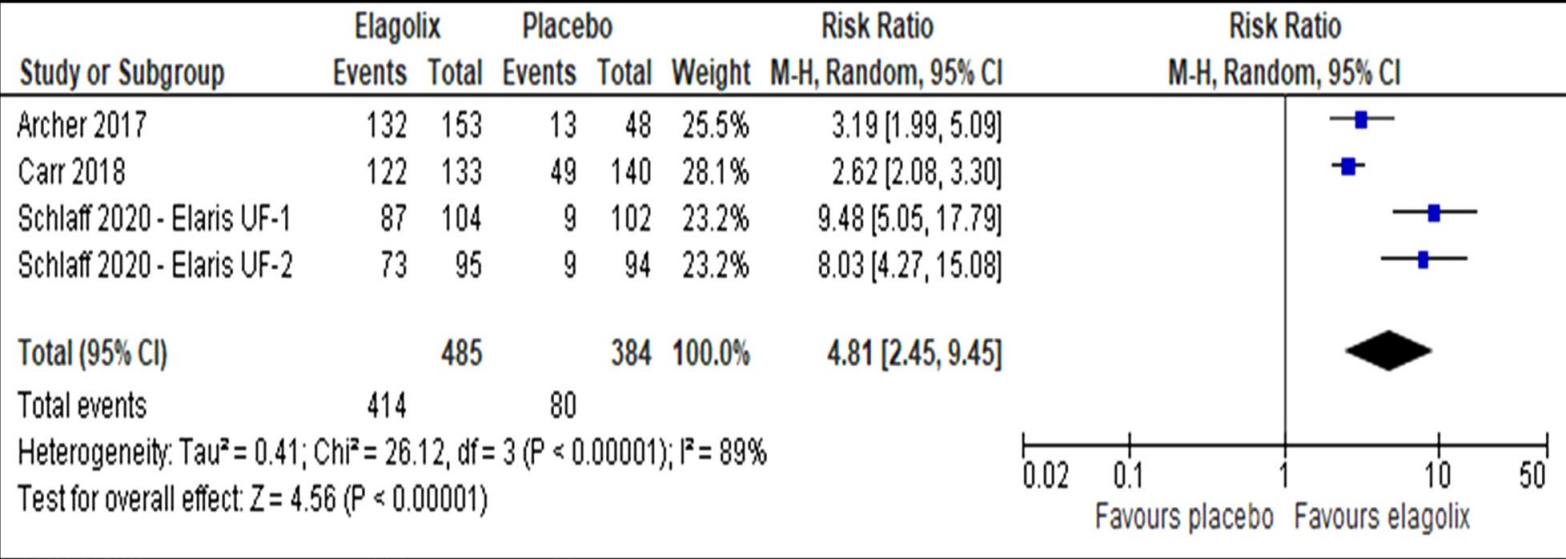
Comparison between elagolix and placebo for the outcome reduction of menstrual blood loss of less than 80 ml

**Fig. 5:**
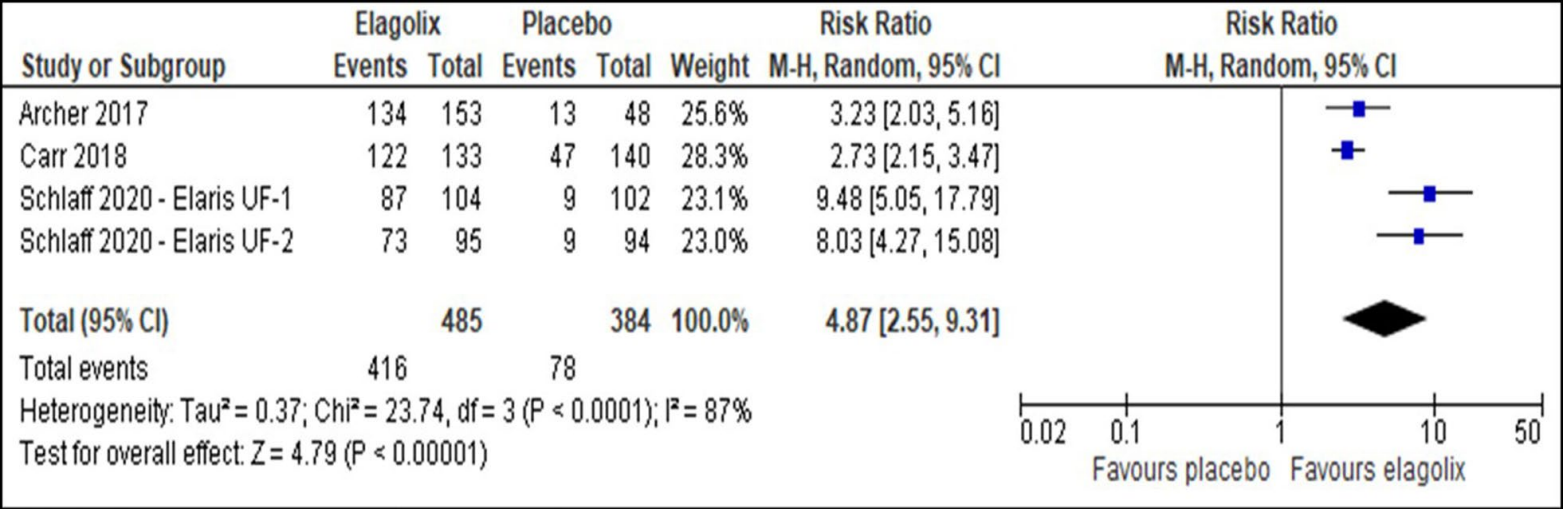
Comparison between elagolix and placebo for the outcome reduction of menstrual blood loss of more than 50%

Elagolix has increased the number of patients with a reduction of menstrual blood loss of less than 80 ml (RR 4.81, 95% CI 2.45 to 9.45; I^2^ statistic = 89%; *P* < 0.001; four trials, 869 participants; moderate quality evidence) (Fig. 4, Table 2) [27–29] or more than 50% from baseline (RR 4.87, 95% CI 2.55 to 9.31; I^2^ statistic = 87%; *P* < 0.001; four trials, 869 participants; moderate quality evidence) (Fig. 5, Table 2) [27–29] compared to placebo. The sensitivity analysis did not change the cumulative effect estimate. Table 3 showed the subgroup analysis for reduction of menstrual blood loss of less than 80 ml or more than 50% reduction from baseline stratified by frequency of drug administration, uterine and fibroid volume (Additional file 1).

**Table 2:** Summary of findings including GRADE quality assessment for comparison between elagolix and placebo

Certainty assessment							No of patients		Effect		Certainty
No of studies	Study design	Risk of bias	Inconsistency	Indirectness	Imprecision	Other consideration	Elagolix	Placebo	Relative (95% CI)	Absolute (95% CI)	
*Reduction of menstrual blood loss of less than 80 ml*											
4	Randomized trials	Not serious ^a^	Serious ^b^	Not serious	Not serious	None	414/485 (85.4%)	80/834 (20.8%)	RR 4.81 (2.45 to 9.45)	794 more per 1000 (from 302 more to 1,000 more)	⨁⨁⨁◯ MODERATE
*Reduction of more than 50% menstrual blood loss*											
4	Randomized trials	Not serious	Serious ^c^	Not serious	Not serious	None	416/485 (85.8%)	78/384 (20.3%)	RR 4.87 (2.55 to 9.31)	362 more per 1000 (from 231 to 528 more)	⨁⨁⨁◯ MODERATE
*Improvement in hemoglobin level*											
4	Randomized trials	Not serious	Not serious	Not serious	Serious	None	196/320 (61.3%)	58/234 (24.8%)	RR 2.46 (1.93 to 3.13)	786 more per 1000 (from 315 to 1000 more)	⨁⨁⨁◯ MODERATE
*Adverse event (Hot flush)*											
4	Randomized trials	Not serious	Not serious ^e^	Not serious	Serious ^d^	None	259/501 (51.7%)	25/389 (6.4%)	RR 7.47 (4.99 to 11.18)	416 more per 1000 (from 256 to 654 more)	⨁⨁⨁◯ MODERATE
*Uterine volume*											
4	Randomized trials	Not serious	Serious ^f^	Not serious	Not serious	None	424	359	–	MD 34.5 lower (43.48 lower to 25.53 lower)	⨁⨁⨁◯ MODERATE
*Fibroid volume*											
4	Randomized trials	Not serious	Serious ^g^	Not serious	Not serious	None	406	344	–	MD 31.39 lower (44.69 lower to 18.09 lower)	⨁⨁⨁◯ MODERATE
*Symptom severity*											
4	Randomized trials	Not serious	very serious ^h^	Not serious	Not serious	None	445	369	–	MD 31.54 lower (41.85 lower to 21.22 lower)	⨁⨁◯◯ LOW
*Health-related quality of life*											
4	Randomized trials	Not serious	Very serious	Not serious	Not serious	None	443	369	–	MD 30.64 higher (20.14 higher to 41.15 higher)	⨁⨁◯◯ LOW
*Bone mineral density (Lumbar spine)*											
3	Randomized trials	Not serious	Not serious	Not serious	Serious	None	281	293	–	MD 2.82 lower (3.3 lower to 2.35 lower)	⨁⨁⨁◯ MODERATE
*Bone mineral density (Total hip)*											
3	Randomized trials	Not serious	Not serious	Not serious	Serious	None	281	293	-	MD 1.97 lower (2.37 lower to 1.57 lower)	⨁⨁⨁◯ MODERATE
*Bone mineral density (Femoral neck)*											
3	Randomized trials	Not serious	Not serious	Not serious	Serious	None	281	293	–	MD 1.92 lower (2.61 lower to 1.23 lower)	⨁⨁⨁◯ MODERATE

**Table 3:** Summary of findings, including GRADE quality assessment for the comparison between elagolix and placebo by subgroup analysis

Outcome/Subgroup		No of trials	No of participants	Risk Ratio (RR)	95% Confidence interval (CI)	P value	Random effect; I^2^ statistic (%)	GRADE quality
*Reduction of menstrual blood loss of less than 80 ml*								
Frequency of drug administration	Twice a day (bd)	4	663	4.90	2.59, 9.25	*P* < 0.001	84	Low
	Once a day (qd)	2	223	3.35	1.28, 8.78	*P* = 0.010	59	Low
Uterine volume	< 500 cm^3^	2	311	8.75	4.97, 15.42	*P* < 0.001	0	Moderate
	> 500 cm^3^	3	558	3.66	1.96, 6.83	*P* < 0.001	84	Very low
Fibroid volume	< 50 cm^3^	2	252	8.77	4.98, 15.45	*P* < 0.001	0	Moderate
	> 50 cm^3^	3	632	3.85	2.09, 7.09	*P* < 0.001	84	Very low
*Reduction of more than 50% menstrual blood loss*								
Frequency of drug administration	Twice a day (bd)	4	663	5.00	2.74, 9.13	*P* < 0.001	82	Low
	Once a day (qd)	2	221	2.47	1.87, 3.26	*P* < 0.001	0	Moderate
Uterine volume	< 500 cm^3^	2	311	8.75	4.97, 15.42	*P* < 0.001	0	Moderate
	> 500 cm^3^	3	558	3.75	2.06, 6.82	*P* < 0.001	82	Very low
Fibroid volume	< 50 cm^3^	2	252	4.66	0.92, 21.71	*P* = 0.060	92	Very low
	> 50 cm^3^	3	632	3.92	2.19, 7.03	*P* < 0.001	82	Very low

For the secondary outcomes, elagolix has increased the number of patients with improved hemoglobin level (RR 2.46, 95% CI 1.93 to 3.13; I^2^ statistic = 0%; *P* < 0.001; four trials, 554 participants; moderate quality evidence) [27–29], reduced the mean percentage change in uterine volume (MD − 34.50, 95% CI − 43.48 to − 25.53; I^2^ statistic = 63%; *P* < 0.001; four trials, 783 participants; moderate quality evidence) [27–29], fibroid volume (MD − 31.39, 95% CI − 44.69 to − 18.09; I^2^ statistic = 65%; *P* < 0.001; four trials, 750 participants; moderate quality evidence) [27–29], severity of symptoms (MD − 31.54, 95% CI − 41.85 to − 21.22; I^2^ statistic = 96%; *P* < 0.001; four trials, 814 participants; low quality evidence) [27–29], and improved health-related quality of life (MD 30.64, 95% CI 20.14 to 41.15; I^2^ statistic = 95%; *P* < 0.001; four trials, 812 participants; low quality evidence) [27–29] (Additional file 1, Table 2) compared to placebo.

**Fig. 6:**
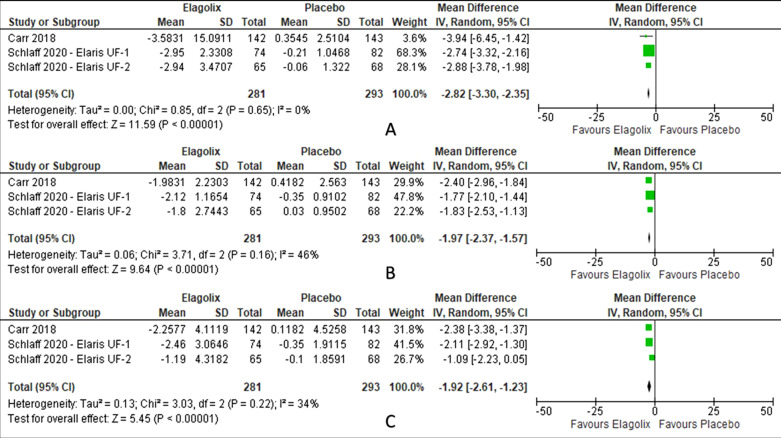
Comparison between elagolix and placebo for the outcome of bone mineral density (**A**: lumbar spine, **B**: total hip, **C**: femoral neck)

Elagolix has reduced bone mineral density in lumbar spine (MD − 2.82, 95% CI − 3.30 to − 2.35; I^2^ statistic = 0%; *P* < 0.001; three trials, 574 participants; moderate quality evidence) [28, 29], total hip (MD − 1.97, 95% CI − 2.37 to − 1.57; I^2^ statistic = 46%; *P* < 0.001; three trials, 574 participants; moderate quality evidence) [28, 29] and femoral neck (MD − 1.92, 95% CI − 2.61 to − 1.23; I^2^ statistic = 34%; *P* < 0.001; three trials, 574 participants; moderate quality evidence) [28, 29] (Fig. 6, Table 2) compared to placebo.

**Fig. 7:**
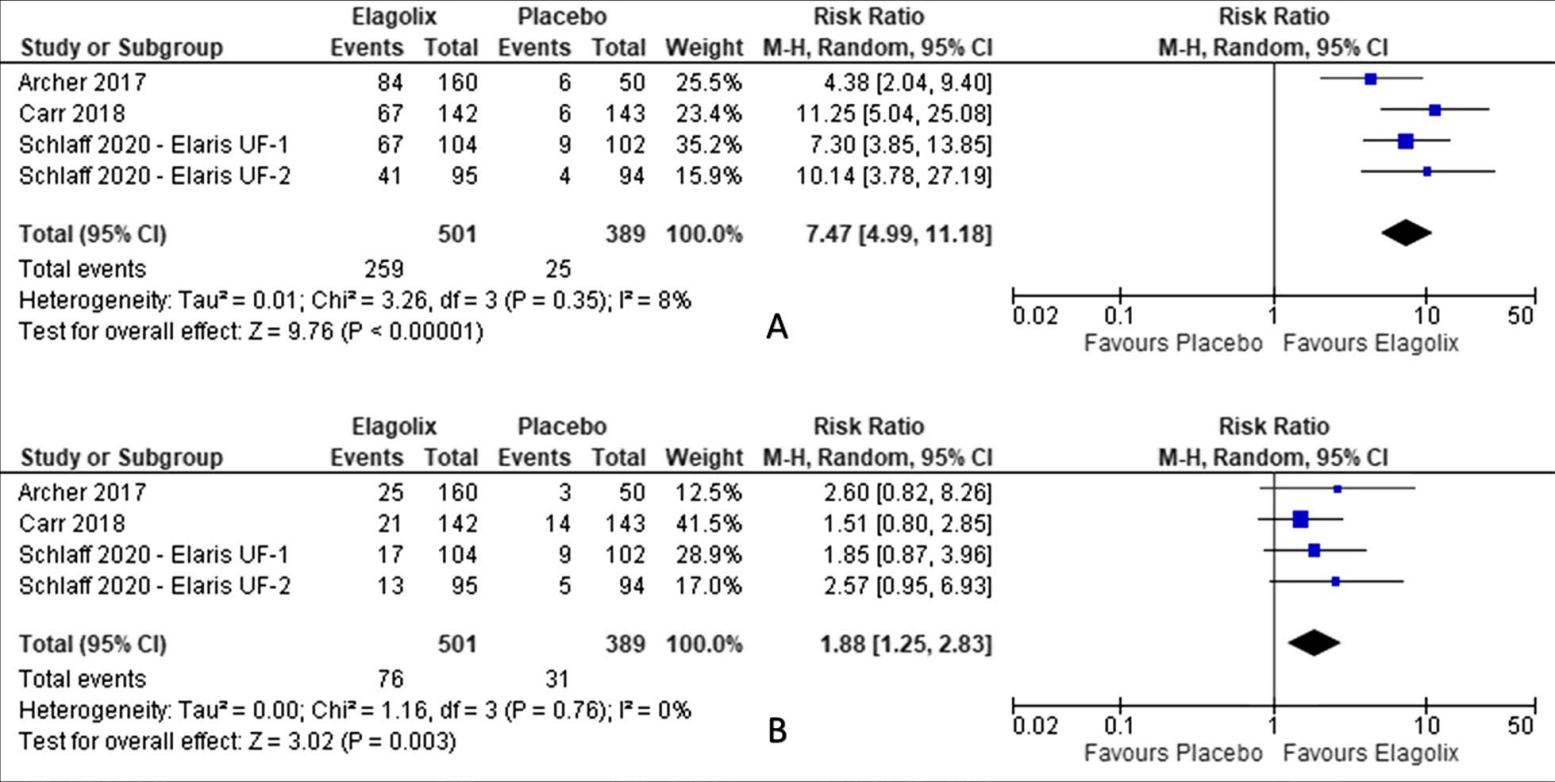
Comparison between elagolix and placebo for the outcome of adverse events (**A**: hot flush, **B**: headache)

There was no significant of severe, serious or adverse event led to discontinuation of elagolix treatment. Elagolix has increased the number of patients with side effect of hot flush (RR 7.47, 95% CI 4.99 to 11.18; I^2^ statistic = 8%; *P* < 0.001; four trials, 890 participants; moderate quality evidence) [27–29] and headache (RR 1.88, 95% CI 1.25 to 2.83; I^2^ statistic = 0%; *P* < 0.001; four trials, 890 participants; low quality evidence) [27–29] (Fig. 7, Table 4) compared to placebo.

**Table 4:** Summary of findings, including GRADE quality assessment for the comparison between elagolix and placebo by adverse events

Adverse event	No of trials	No of participants	Risk Ratio (RR)	95% Confidence interval (CI)	*P* value	Random effect; I^2^ statistic (%)	GRADE quality
Any AE	4	890	1.25	1.15, 1.36	*P* < 0.001	0	High
Serious AE	4	890	0.93	0.48, 1.81	*P* = 0.830	0	Low
Severe AE	3	605	1.53	0.86, 2.73	*P* = 0.150	0	Low
AE led to discontinuation	4	890	1.66	1.05, 2.64	*P* = 0.030	0	Low
Hot flush	4	890	7.47	4.99, 11.18	*P* < 0.001	8	Moderate
Headache	4	890	1.88	1.25, 2.83	*P* = 0.003	0	Low
Abdominal pain	2	495	1.17	0.37, 3.66	*P* = 0.790	6	Low
Dizziness	2	495	1.26	0.48, 3.29	*P* = 0.640	18	Low
Nausea	4	890	1.00	0.53, 1.92	*P* = 0.990	41	Low
Fatigue	4	890	0.77	0.33, 1.79	*P* = 0.550	0	Low
Hypertension	2	495	1.25	0.14, 10.93	*P* = 0.840	*	Low

^*^Not estimable due to no hypertension events for both Elagolix and placebo. Carr et al., 2018

B) Comparison between elagolix and elagolix with estradiol/norethindrone acetate.

**Fig. 8:**
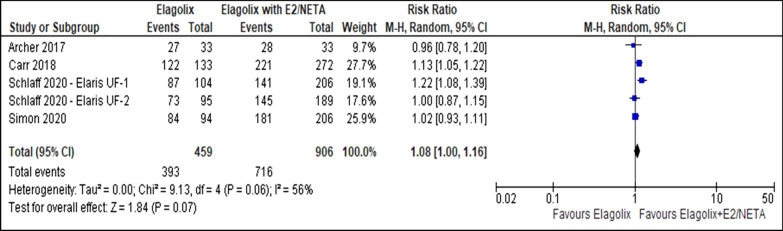
Comparison between elagolix and elagolix with estradiol/norethindrone acetate for the outcome reduction of menstrual blood loss of less than 80 ml

**Fig. 9:**
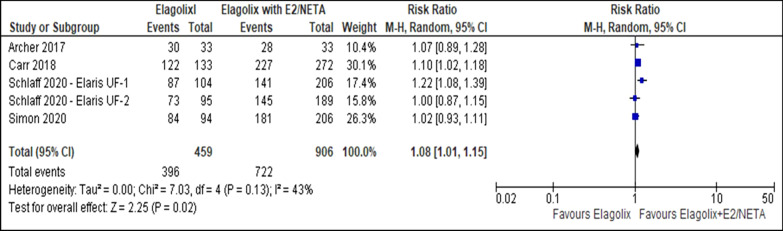
Comparison between elagolix and elagolix with estradiol/norethindrone acetate for the outcome reduction of more than 50% menstrual blood loss

There was no difference in menstrual blood loss of less than 80 ml (RR 1.08, 95% CI 1.00 to 1.16; I^2^ statistic = 56%; *P* = 0.070; five trials, 1365 participants; moderate quality evidence) (Fig. 8, Table 5) [27–30] or more than 50% reduction from baseline between the elagolix (RR 1.08, 95% CI 1.01 to 1.15; I^2^ statistic = 43%; *P* = 0.020; five trials, 1365 participants; high quality evidence) (Fig. 9, Table 5) [27–30] and elagolix with estradiol/norethindrone acetate. The sensitivity analysis did not change the cumulative effect estimate. Table 6 showed the subgroup analysis for reduction of menstrual blood loss of less than 80 ml or more than 50% reduction from baseline stratified by dosage and uterine volume (Additional file 1).

**Table 5:** Summary of findings, including GRADE quality assessment for the comparison between elagolix and elagolix with estradiol/norethindrone acetate

Certainty assessment							No of patients		Effect		Certainty
No of studies	Study design	Risk of bias	Inconsistency	Indirectness	Imprecision	Other consideration	Elagolix	Elagolix with estradiol plus norethindrone acetate	Relative (95% CI)	Absolute (95% CI)	
*Reduction of menstrual blood loss less than 80 ml*											
5	Randomized trials	Not serious	Serious	Not serious	Not serious	None	393/459 (85.6%)	716/906 (79.0%)	RR 1.08 (1.00 to 1.16)	63 more per 1,000 (from 0 fewer to 126 more)	⨁⨁⨁◯ MODERATE
*Reduction of more than 50% menstrual blood loss*											
5	Randomized trials	Not serious	Not serious	Not serious	Not serious	None	396/459 (86.3%)	722/906 (79.7%)	RR 1.08 (1.01 to 1.15)	64 more per 1,000 (from 8 to 120 more)	⨁⨁⨁⨁ HIGH
*Improvement in hemoglobin level*											
5	Randomized trials	Not serious	Serious	Not serious	Serious	None	206/319 (64.6%)	370/580 (63.8%)	RR 0.99 (0.80 to 1.22)	6 fewer per 1,000 (from 128 fewer to 140 more)	⨁⨁◯◯ LOW
*Adverse event (Hot flush)*											
5	Randomized trials	Not serious	Not serious	Not serious	Serious ^a^	None	258/474 (54.4%)	190/929 (20.5%)	RR 2.67 (2.30 to 3.10)	342 more per 1000 (from 266 to 429 more)	⨁⨁⨁◯ MODERATE
*Uterine volume*											
5	Randomized trials	Not serious	Serious ^b^	Not serious	Not serious	None	422	828	–	MD 17.47 lower (27.54 lower to 7.4 lower)	⨁⨁⨁◯ MODERATE
*Fibroid volume*											
5	Randomized trials	Not serious	Not serious	Not serious	Not serious	None	408	800	–	MD 23.18 lower (28.98 lower to 17.38 lower)	⨁⨁⨁⨁ HIGH
*Symptom severity*											
5	Randomized trials	Not serious	Not serious	Not serious	Not serious	None	429	859	–	MD 9.05 lower (9.68 lower to 8.43 lower)	⨁⨁⨁⨁ HIGH
*Health-related quality of life*											
5	Randomized trials	Not serious	Very serious ^c^	Not serious	Not serious	None	428	859	–	MD 9.94 higher (5.82 higher to 14.06 higher)	⨁⨁◯◯ LOW
*Bone mineral density (Lumbar spine)*											
4	Randomized trials	Not serious	Not serious	Not serious	Serious	none	362	764	–	MD 2.63 lower (3.12 lower to 2.14 lower)	⨁⨁⨁◯ MODERATE
*Bone mineral density (Total hip)*											
4	Randomized trials	Not serious	Not serious	Not serious	Serious	None	362	764	–	MD 1.93 lower (2.56 lower to 1.31 lower)	⨁◯◯◯ VERY LOW
*Bone mineral density (Femoral neck)*											
4	Randomized trials	Not serious	Not serious	Not serious	Serious	None	362	764	–	MD 0.77 lower (1.84 lower t0 0.3 higher)	⨁◯◯◯ VERY LOW

**Table 6:** Summary of findings, including GRADE quality assessment for the comparison between elagolix and elagolix with estradiol/norethindrone acetate by subgroup analysis

Outcome/Subgroup		No of trials	No of participants	Risk Ratio (RR)	95% Confidence interval (CI)	P value	Random effect; I^2^ statistic	GRADE quality
*Reduction of menstrual blood loss of less than 80 ml*								
Dosage of E2/NETA	0.5 mg E2/ 0.1 mg NETA	2	333	1.08	0.92, 1.27	*P* = 0.350	52%	Moderate
	1.0 mg E2/ 0.5 mg NETA	4	1165	1.08	1.00, 1.18	*P* = 0.060	58%	Moderate
Uterine volume	< 500 cm^3^	3	894	1.07	0.95, 1.21	*P* = 0.250	70%	Low
	> 500 cm^3^	2	471	1.08	0.94, 1.24	*P* = 0.290	46%	Moderate
*Reduction of more than 50% menstrual blood loss*								
Dosage of E2/NETA	0.5 mg E2/ 0.1 mg NETA	2	333	1.10	1.01, 1.19	*P* = 0.020	0%	Moderate
	1.0 mg E2/ 0.5 mg NETA	4	1165	1.08	0.99, 1.17	*P* = 0.070	56%	Moderate
Uterine volume	< 500 cm^3^	3	894	1.07	0.95, 1.21	*P* = 0.250	70%	Low
	> 500 cm^3^	2	471	1.10	1.02, 1.17	*P* = 0.009	0%	Moderate

E2—estradiol; NETA—norethindrone acetate

Foe secondary outcomes, there was no difference improvement in hemoglobin level between elagolix (RR 0.99, 95% CI 0.80 to 1.22; I^2^ statistic = 68%; *P* = 0.930; five trials, 899 participants; low quality evidence) [27–30] and elagolix with estradiol/norethindrone acetate. However, elagolix has reduced mean percentage change in uterine volume (MD − 17.47, 95% CI − 27.54 to − 7.40; I^2^ statistic = 58%; *P* < 0.001; five trials, 1250 participants; moderate quality evidence) [27–30], fibroid volume (MD − 23.18, 95% CI − 28.98 to − 17.38; I^2^ statistic = 0%; *P* < 0.001; five trials, 1208 participants; high quality evidence) [27–30], symptoms severity (MD − 9.05, 95% CI − 9.68 to − 8.43; I^2^ statistic = 0%; *P* < 0.001; five trials, 1288 participants; high quality evidence) [27–30], and increased health-related quality of life (MD 9.94, 95% CI 5.82 to 14.06; I^2^ statistic = 76%; *P* < 0.001; five trials, 1287 participants; low quality evidence) [27–30] (Additional file 1, Table 5) compared to elagolix with estradiol/norethindrone acetate.

**Fig. 10:**
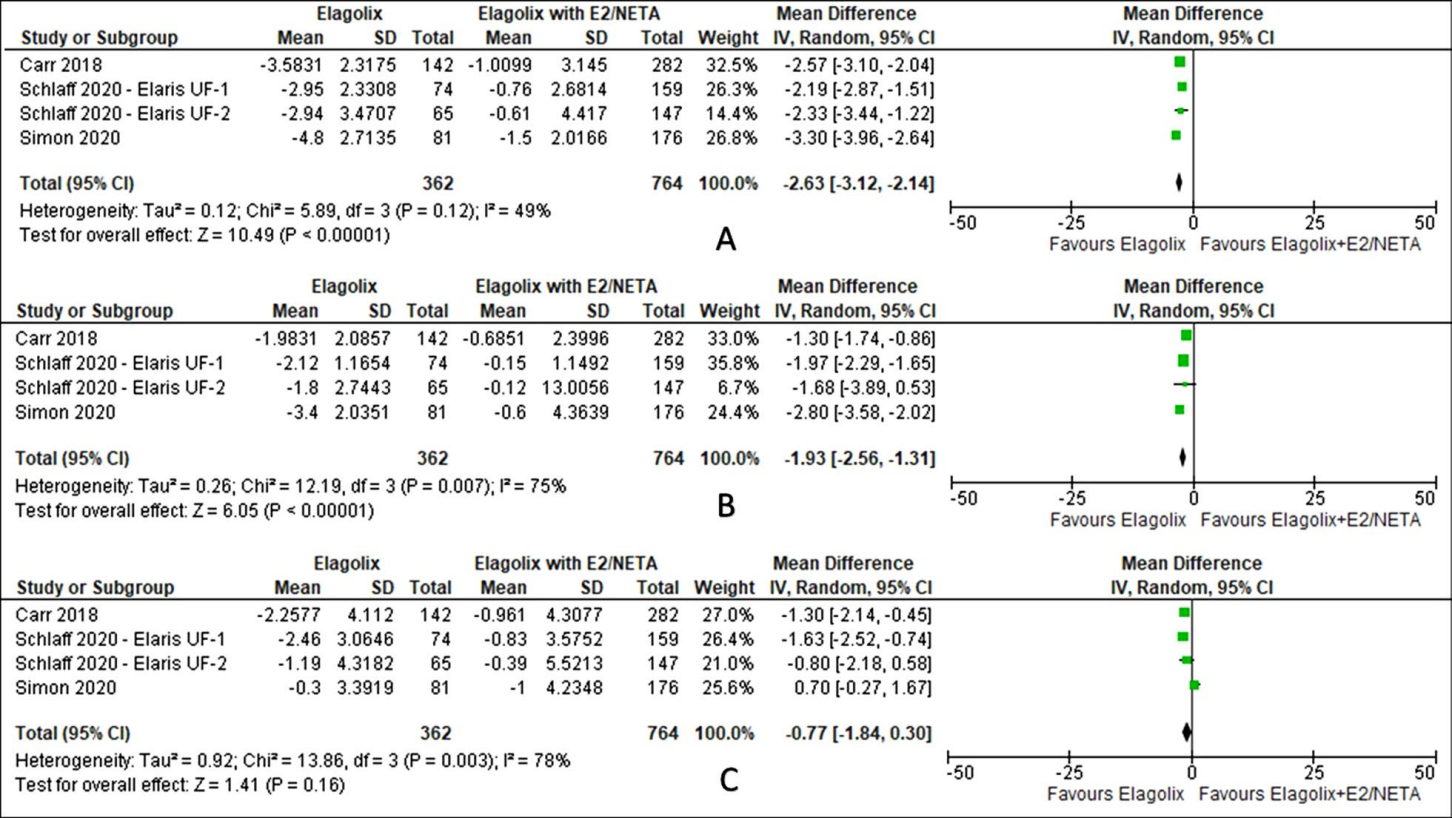
Comparison between elagolix and elagolix with estradiol/norethindrone acetate for the outcome of bone mineral density (**A**: lumbar spine, **B**: total hip, **C**: femoral neck)

Elagolix has reduced bone mineral density in the lumbar spine (MD − 2.63, 95% CI − 3.12 to − 2.14; I^2^ statistic = 49%; *P* < 0.001; four trials, 1126 participants; moderate quality evidence [28–30], and total hip (MD − 1.93, 95% CI − 2.56 to − 1.31; I^2^ statistic = 75%; *P* < 0.001; four trials, 1126 participants; very low quality evidence) [28–30] except femoral neck (MD − 0.77, 95% CI − 1.84 to 0.30; I^2^ statistic = 78%; *P* = 0.160; four trials, 1126 participants; very low quality evidence) [28–30] (Fig. 10, Table 5) compared to elagolix with estradiol/norethindrone acetate.

**Fig. 11:**
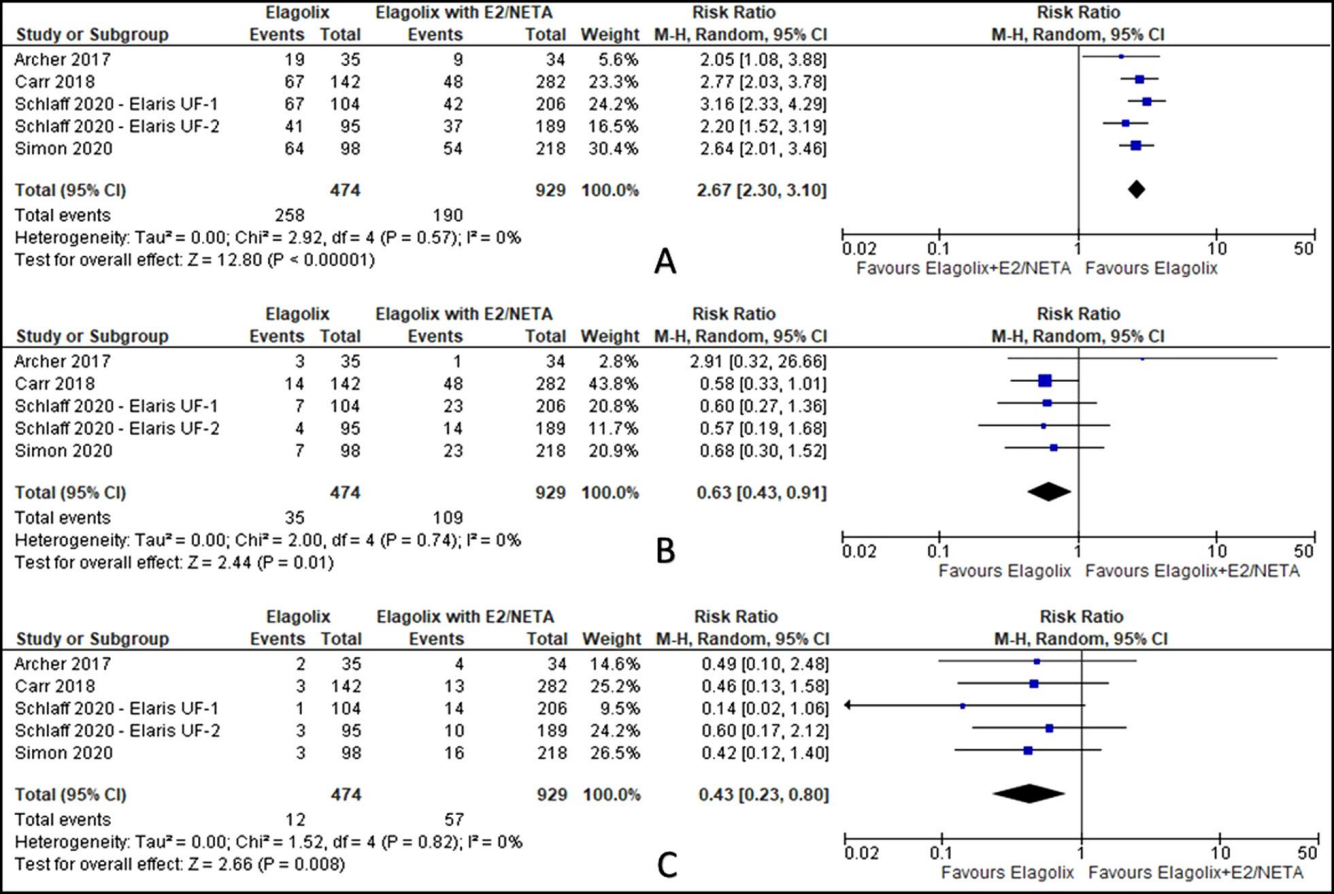
Comparison between elagolix and elagolix with estradiol/norethidrone acetate for the outcome adverse event (**A**: hot flush, **B**: nausea, **C**: fatigue)

There was no difference of severe, serious or adverse event led to discontinuation between elagolix treatment and elagolix with estradiol/norethindrone acetate. Elagolix has increased the number of patients with side effect of hot flush (RR 2.67, 95% CI 2.30 to 3.10; I^2^ statistic = 0%; *P* < 0.001; five trials, 1403 participants; moderate quality evidence) [27–30], reduced the number of patients with risk of nausea (RR 0.63, 95% CI 0.43 to 0.91; I^2^ statistic = 0%; *P* = 0.010; five trials, 1403 participants; low quality evidence) [27–30] and fatigue (RR 0.43, 95% CI 0.23 to 0.80; I^2^ statistic = 0%; *P* = 0.008; five trials, 1403 participants; low quality evidence) [27–30] (Fig. 11, Table 7) compared to elagolix with estradiol/norethindrone acetate.

**Table 7:** Summary of findings, including GRADE quality assessment for comparison between elagolix and elagolix with estradiol/norethindrone acetate by adverse events

Adverse event	No of trials	No of participants	Risk Ratio (RR)	95% Confidence interval (CI)	*P* value	Random effect; I^2^ statistic (%)	GRADE quality
Any AE	5	1403	1.13	1.03, 1.25	*P* = 0.010	68	Moderate
Serious AE	5	1403	1.23	0.68, 2.24	*P* = 0.500	0	Low
Severe AE	4	979	0.90	0.45, 1.83	*P* = 0.780	51	Low
AE led to discontinuation	5	1403	1.31	0.92, 1.87	*P* = 0.130	0	Low
Hot flush	5	1403	2.67	2.30, 3.10	*P* < 0.001	0	Moderate
Headache	5	1403	1.16	0.84, 1.62	*P* = 0.370	22	Low
Abdominal pain	2	493	1.02	0.14, 7.47	*P* = 0.990	47	Low
Dizziness	2	493	0.87	0.38, 2.02	*P* = 0.750	0	Low
Nausea	5	1403	0.63	0.43, 0.91	*P* = 0.010	0	Low
Fatigue	5	1403	0.43	0.23, 0.80	*P* = 0.008	0	Low
Hypertension	3	809	0.60	0.23, 1.59	*P* = 0.300	0	Low

## Discussion

### Main findings

This review was designed to include all randomized controlled trials that addressed the efficacy of elagolix treatment in women with heavy menstrual blood loss associated with uterine fibroid. The four identified trials formed comparisons either with placebo or with elagolix and estradiol/norethindrone acetate. The result showed that elagolix treatment increased the number of patients who had menstrual blood loss of less than 80 ml or more than 50% reduction from baseline compared to placebo. However, there was no difference when elagolix was combined with estradiol/norethindrone acetate. Elagolix treatment also had reduced the mean percentage change in both fibroid and uterine volume in both comparisons.

The review showed more patients with improved hemoglobin level in elagolix treatment than placebo, but there was no difference in elagolix with estradiol/norethindrone acetate group. Elagolix also has reduced the severity of symptoms and increased the health-related quality of life in both comparisons. Nevertheless, more patients had adverse events such as hot flush, headache, and bone mineral density loss compared to placebo. Still, these hypoestrogenic effects were attenuated with the addition of estradiol/norethindrone acetate. In the subgroup analysis by dosage, frequency of drug administration, uterine volume, and fibroid volume, the high heterogeneity cannot be explained but has vanished in uterine volume < 500 cm^3^, fibroid volume < 50 cm^3^, and low dose estradiol/norethindrone acetate.

There were other two reviews in this regard, including one on predictors of response to elagolix with estradiol/norethindrone acetate and the other on medical treatment of uterine fibroid [4, 23]. Al-Hendy 2020 looked at independent variables of only one trial [29]. This review found that elagolix with estradiol/norethindrone acetate successfully reduced heavy menstrual bleeding caused by uterine fibroids regardless of patients’ age, body mass index, race, ethnicity, baseline menstrual blood loss, fibroid location, or uterine and primary fibroid volume. Sabry 2012 had reviewed the hormonal and nonhormonal treatment of uterine fibroid. Our review included three additional trials [27, 28, 30]. All four trials were related to our prespecified primary and secondary outcomes. The secondary outcome focused on bone mineral density loss, hemoglobin level improvement, symptoms’ severity, and health-related quality of life, which are not covered in Al-Hendy 2020. The current review also focused on the hypoestrogenism side effect of elagolix that is attenuated with estradiol/norethindrone acetate.

### Limitations

We had performed a comprehensive literature review to assess the effectiveness and role of elagolix in reducing heavy menstrual blood associated with uterine fibroid. We included four trials, but the results could apply to premenopausal women. Only one trial looked at the effects of elagolix over twelve months [30]. Thus, the results of this study are limited in their applicability for long-term care. There were also insufficient trials for elagolix dose subgroup review. However, most trials used the formulation elagolix 300 mg bd and 600 mg qd (total 600 mg daily). In all probability, these dosage forms can be used as therapy.

Elagolix had a good efficacy profile except for its hypoestrogenism side effects of hot flush, headache, and bone mineral density loss. However, these side effects can be reduced by combination with estradiol/norethindrone acetate. Women who are at risk of osteoporosis or on long-term prednisolone treatment may benefit from combination formulation therapy with no serious or life-threatening side effects.

The quality of trial evidence was variable. Generally, there was a low or unclear risk of bias for most trials in most domains. There was no evidence of selective reporting bias. The lack of adequate random sequence generation can lead to treatment effect bias in the original study and the subsequent review. All four trials were funded by AbbVie pharmaceutical. We had encountered moderate and high heterogeneity in most of our meta-analyses. The sensitivity analysis did not change the cumulative effect estimate. Some outcomes showed substantial heterogeneity. Therefore, the overall level of evidence contributing to this review is moderate to low quality. There was also a wide variation in the frequency of adverse events reported in the included studies due to definitions differences, difficulty in identifying and reporting adverse events.

We attempted to reduce publication bias by checking the reference lists of all related studies for further references and searching multiple databases without language restriction. However, we cannot be certain that we have located all the trials in this area. Since we have only four included trials, we could not construct a funnel plot for detecting bias or heterogeneity due to insufficient studies. All included trials had reported approximately almost all outcomes. Treatment periods differed in two trials [27, 30] but the outcome was unlikely to be influenced. The outcome was unlikely to be affected, although the process of randomization and allocation concealment were not stated in all trials.

All four included trials were funded and prospectively registered under clinicaltrial.gov. The primary outcome was measured using the well-established alkaline hematin method. Although all the studies showed the same direction of effect, we encountered moderate heterogeneity in our primary outcome. We were not able to explain this in our subgroup analysis. Other secondary outcomes were objectively assessed using standard measurement, for example, ultrasound, UFS-QoL questionnaire, and dual-energy x-ray absorptiometry scans. In two trials, women with asymptomatic anemia and a hemoglobin level of less than 12 g/dl at screening or during the study period were recommended to take iron supplements. We were uncertain whether this could influence the hemoglobin level.

## Conclusions

Elagolix appears to be effective in reducing heavy menstrual bleeding caused by uterine fibroid in premenopausal women. It also has a beneficial effect on uterine and fibroid volume reduction. Furthermore, it reduces the severity of symptoms and improves the health-related quality of life. The hemoglobin level also improved with elagolix treatment, but this needs to be justified as the participants were given hematinic supplements at screening and during the treatment period. There were no severe or life-threatening adverse events that contributed to the discontinuation of the biosafety profile. Elagolix with estradiol/norethindrone acetate was effective in combating the hypoestrogenism side effects of hot flushes, headaches, and bone loss. Therefore, women at risk of osteoporosis should be treated with elagolix and estradiol/norethindrone acetate. As a result of this review, many women may be able to avoid surgical intervention with elagolix treatment, which later helps them preserve their fertility.

Data on the study design, setting, randomization method, and blinding should all be reported during the trial to increase the quality of evidence. If further research is done to look at the use of elagolix for uterine fibroid treatment, they would need to produce similar and longer trials with varying elagolix dosages and fibroid location. It helps with heterogeneity subgroup analysis. The importance of age-based inclusion criteria should be emphasized further. Participants only receive a hematinic supplement if they have moderate to severe or symptomatic anemia, to see actual changes in hemoglobin levels.

## Supplementary Information

**Additional file 1**. Funnel plots of the article performing subgroup analysis by frequency of drug administration, dosage of estradiol/norethindrone acetate, uterine volume, fibroid volume, and secondary outcomes of both comparisons, Search strategy

**Additional file 2**. PRISMA checklist

**Additional file 3**. Outcome data extraction form [27–30]

## Data Availability

The datasets generated during and /or analysed during the current study are available in the [Additional files 1, 2 and 3] repository, (https://doi.org/10.6084/m9.figshare.15034881.v1).

## Abbreviations

GnRH: Gonadotropin-releasing hormone
US FDA: United States Food and Drug Administration
UFS-QoL: Uterine Fibroid Symptom and Quality of Life
