# Supplementary material for: Elagolix treatment in women with heavy menstrual bleeding associated with uterine fibroid: a systematic review and meta-analysis
Source: BMC Womens Health. 2022 Jan 15;22:14. doi: 10.1186/s12905-022-01596-2 (PMC8761302; doi:10.1186/s12905-022-01596-2)
Supplement: Supplementary file 1 — Additional file 1. Funnel plots of the article performing subgroup analysis by frequency of drug administration, dosage of estradiol/norethindrone acetate, uterine volume, fibroid volume, and secondary outcomes of both comparisons, Search strategy [file 12905_2022_1596_MOESM1_ESM.docx]

ADDITIONAL FILE 1 (Appendix):


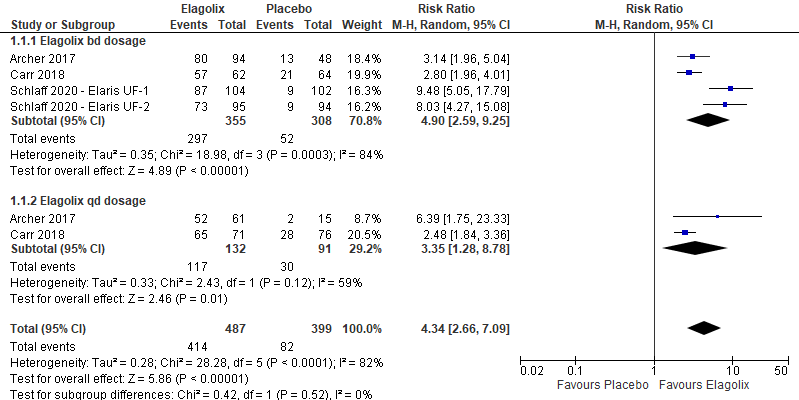


Additional Figure 1: Comparison between elagolix and placebo for the outcome reduction of menstrual blood loss of less than 80 ml by frequency of drug administration.


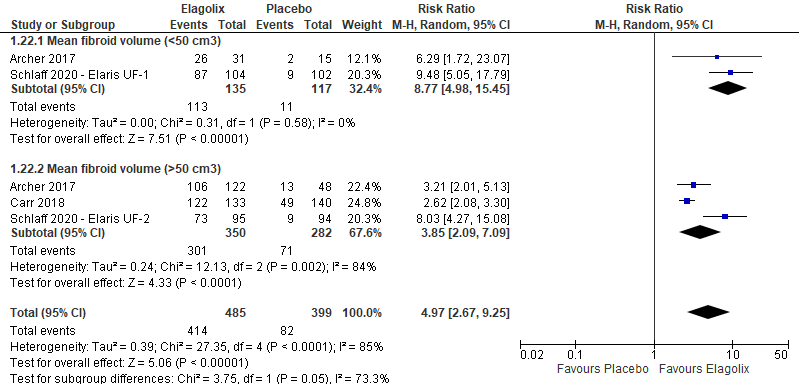


Additional Figure 2: Comparison between elagolix and placebo for the outcome reduction of menstrual blood loss of less than 80 ml by fibroid volume.


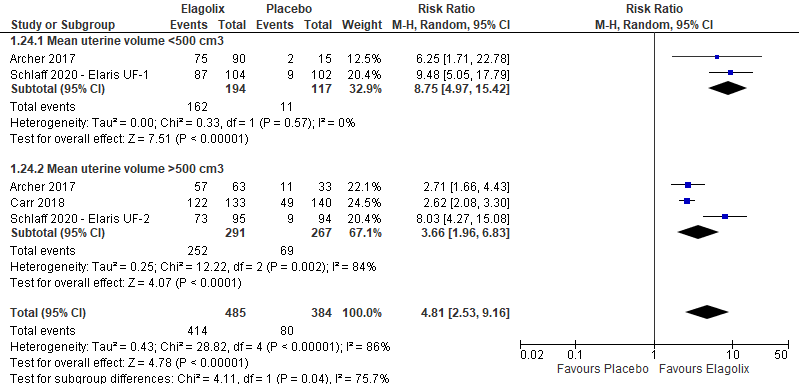


Additional Figure 3: Comparison between elagolix and placebo for the outcome reduction of menstrual blood loss of less than 80 ml by uterine volume.


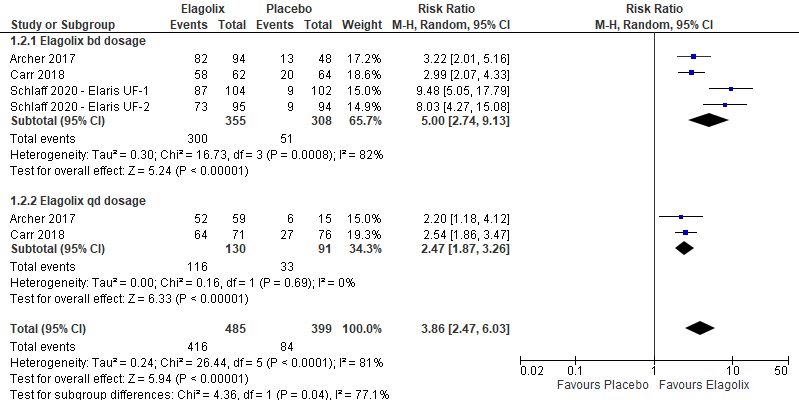


Additional Figure 4: Comparison between elagolix and placebo for the outcome reduction of menstrual blood loss of more than 50% by frequency of drug administration.


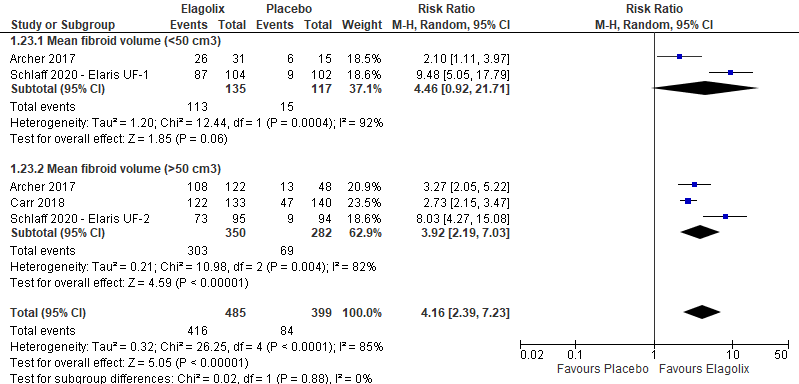


Additional Figure 5: Comparison between elagolix and placebo for the outcome reduction of menstrual blood loss of more than 50% by fibroid volume.


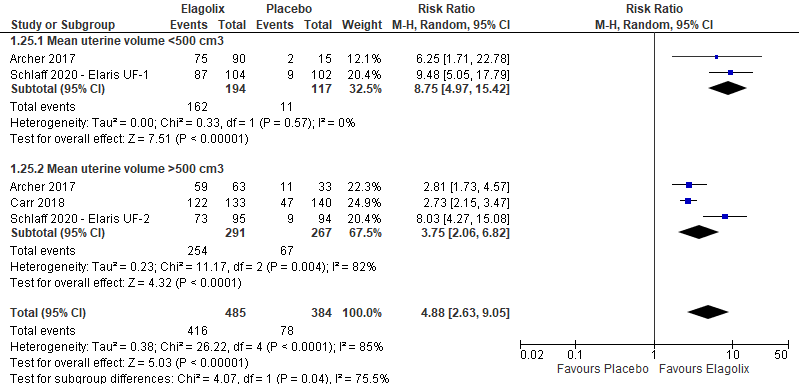
 Additional Figure 6: Comparison between elagolix and placebo for the outcome reduction of menstrual blood loss of more than 50% by uterine volume.


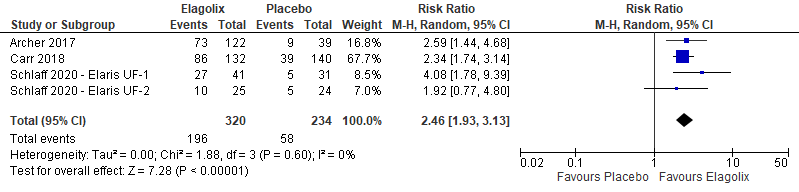
 Additional Figure 7: Comparison between elagolix and placebo for the outcome improvement in hemoglobin level.


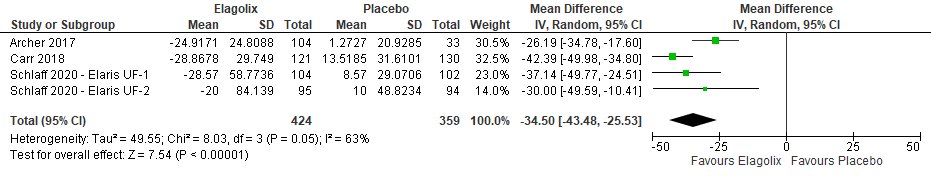
 Additional Figure 8: Comparison between elagolix and placebo for the outcome uterine volume.


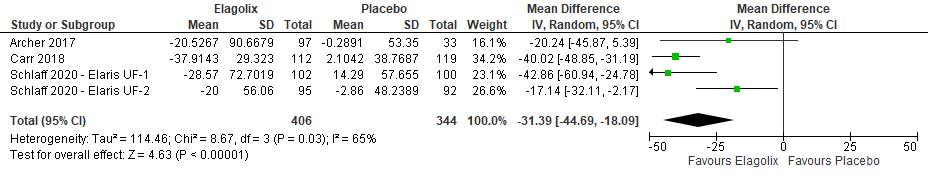
 Additional Figure 9: Comparison between elagolix and placebo for the outcome fibroid volume.


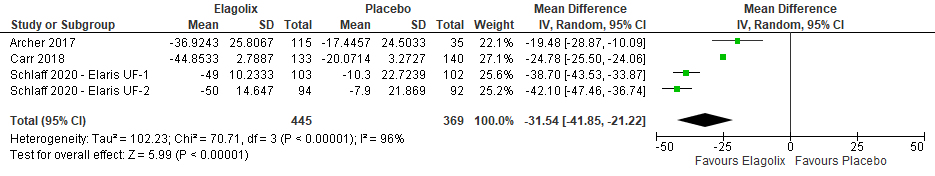
 Additional Figure 10: Comparison between elagolix and placebo for the outcome symptom severity.


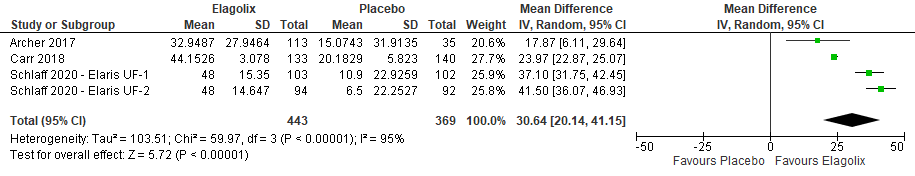


Additional Figure 11: Comparison between elagolix and placebo for the outcome health-related quality of life.


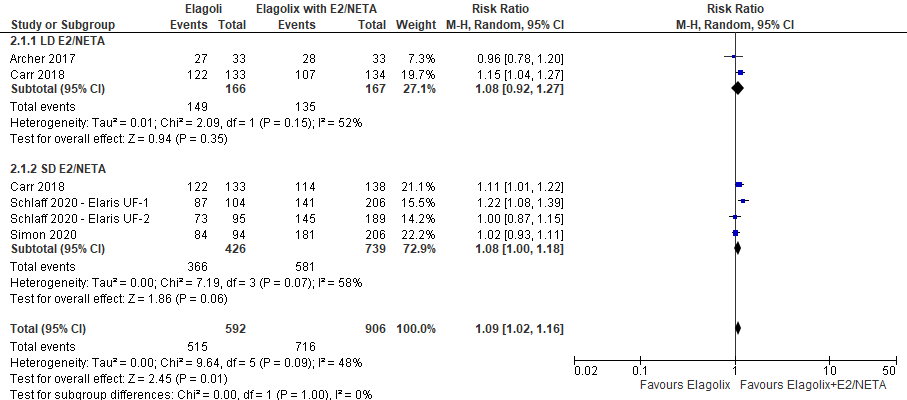


Additional Figure 12: Comparison between elagolix and elagolix with estradiol/norethindrone acetate for the outcome reduction of menstrual blood loss of less than 80 ml by dosage.


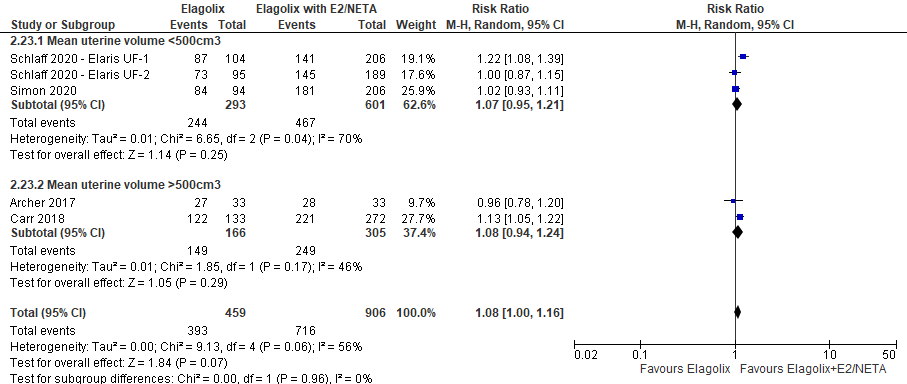


Additional Figure 13: Comparison between elagolix and elagolix with estradiol/norethindrone acetate for the outcome reduction of menstrual blood loss of less than 80 ml by uterine volume.


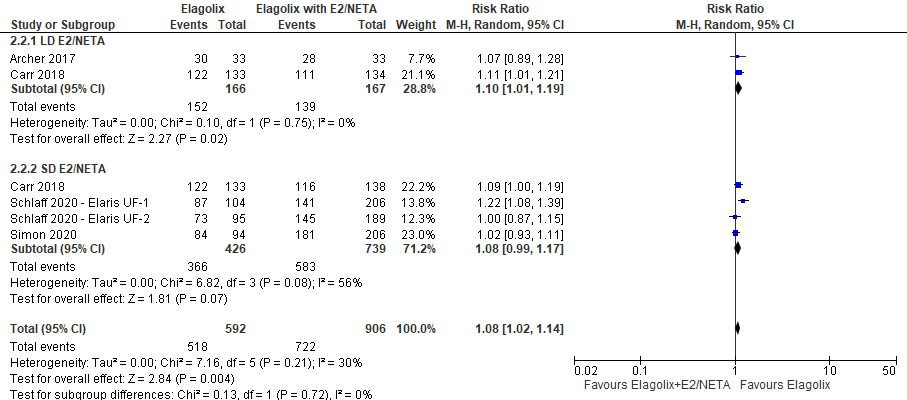


Additional Figure 14: Comparison between elagolix and elagolix with estradiol/norethindrone acetate for the outcome reduction of more than 50% menstrual blood loss by dosage.


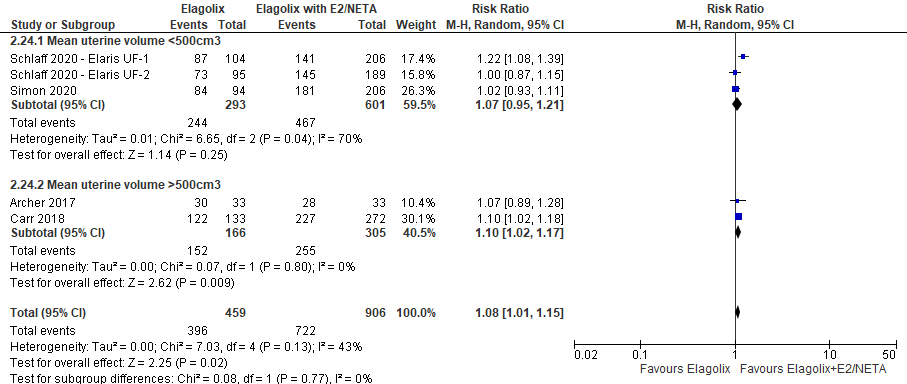


Additional Figure 15: Comparison between elagolix and elagolix with estradiol/norethindrone acetate for the outcome reduction of more than 50% menstrual blood loss by uterine volume.


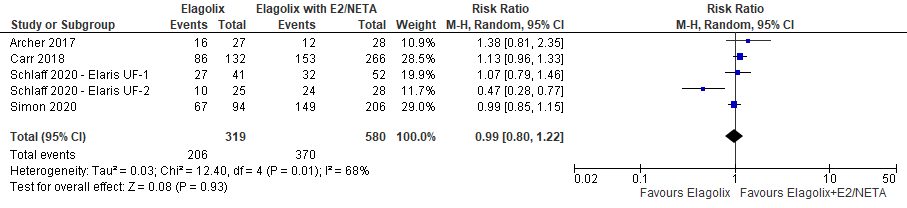


Additional Figure 16: Comparison between elagolix and elagolix with estradiol/norethindrone acetate for the improvement in hemoglobin level.


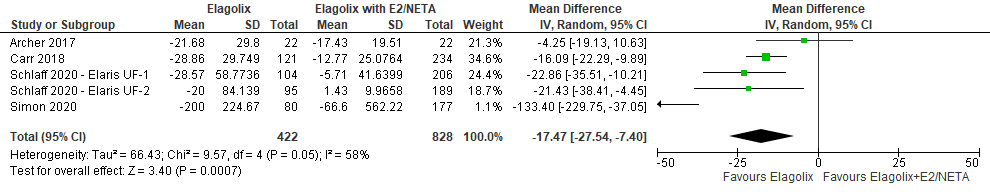


Additional Figure 17: Comparison between elagolix and elagolix with estradiol/norethindrone acetate for the outcome uterine volume.


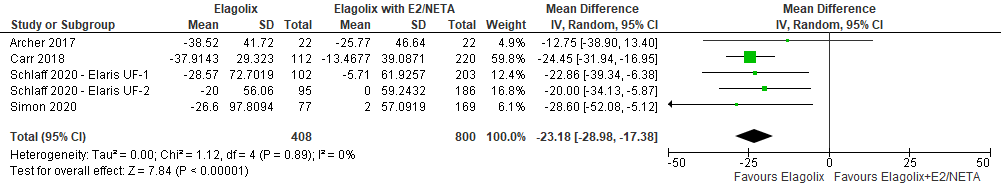


Additional Figure 18: Comparison between elagolix and elagolix with estradiol/norethindrone acetate for the outcome fibroid volume.


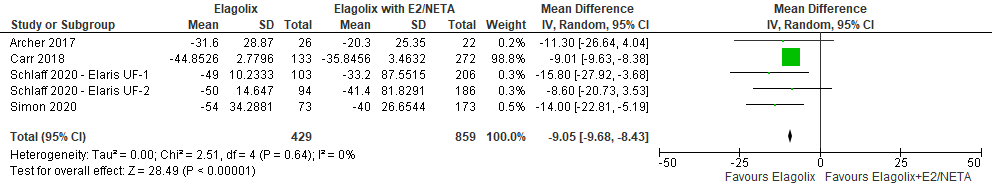


Additional Figure 19: Comparison between elagolix and elagolix with estradiol/norethindrone acetate for the outcome symptoms severity.


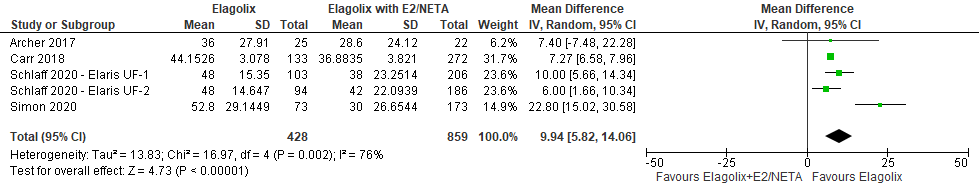


Additional Figure 20: Comparison between elagolix and elagolix with estradiol/norethindrone acetate for the health-related quality of life.

**APPENDIX 1**

**Search strategy**

**CENTRAL, PubMed, ScienceDirect, SCOPUS, EBSCOHOST**

1 leiomyoma

2 fibroid

3 #1 OR #2

4 menorrhagia

5 #3 AND #4

6 ELAGOLIX

7 #5 AND #6

8 "menorrhagia"[MeSH Terms] OR "menorrhagia"[All Fields] OR "menorrhagias"[All Fields] OR ("menorrhagia"[MeSH Terms] OR "menorrhagia"[All Fields] OR ("heavy"[All Fields] AND "menstrual"[All Fields] AND "bleeding"[All Fields]) OR "heavy menstrual bleeding"[All Fields])

9 "elagolix"[Supplementary Concept] OR "elagolix"[All Fields]

10 ((menorrhagia) OR (heavy menstrual bleeding) AND ((ffrft[Filter]) AND (fha[Filter]) AND (randomizedcontrolledtrial[Filter]) AND (fft[Filter]))) AND (elagolix AND ((ffrft[Filter]) AND (fha[Filter]) AND (randomizedcontrolledtrial[Filter]) AND (fft[Filter])))

11 ("menorrhagia"[MeSH Terms] OR "menorrhagia"[All Fields] OR "menorrhagias"[All Fields] OR ("menorrhagia"[MeSH Terms] OR "menorrhagia"[All Fields] OR ("heavy"[All Fields] AND "menstrual"[All Fields] AND "bleeding"[All Fields]) OR "heavy menstrual bleeding"[All Fields])) AND ("loattrfree full text"[Filter] AND "hasabstract"[All Fields] AND "randomized controlled trial"[Publication Type] AND "loattrfull text"[Filter]) AND (("elagolix"[Supplementary Concept] OR "elagolix"[All Fields]) AND ("loattrfree full text"[Filter] AND "hasabstract"[All Fields] AND "randomized controlled trial"[Publication Type] AND "loattrfull text"[Filter]).
