# Supplementary material for: Elagolix treatment in women with heavy menstrual bleeding associated with uterine fibroid: a systematic review and meta-analysis
Source: BMC Womens Health. 2022 Jan 15;22:14. doi: 10.1186/s12905-022-01596-2 (PMC8761302; doi:10.1186/s12905-022-01596-2)
Supplement: Supplementary file 3 — Additional file 3. Outcome data extraction form [27–30] [file 12905_2022_1596_MOESM3_ESM.docx]

***Outcome Data Extraction***

**Dichotomous data**

| **Outcome: Reduction of monthly menstrual blood loss <80 ml** | | | | | |
| --- | --- | --- | --- | --- | --- |
| **Author** | **Intervention group** | | **Control group** | | **Comments** |
|  | Number of participants analyzed | Percentage of participants  (%) | Number of participants analyzed | Percentage of participants  (%) |  |
| (Archer *et al.*, 2017) | 31 | 23/31 (74) | 15 | 2/15 (13) | Cohort 4 Elagolix 100 mg bd (total 200 mg a day) vs Cohort 4 placebo |
| (Archer *et al.*, 2017) | 33 | 28/33 (85) | 18 | 4/18 (22) | Cohort 1 Elagolix 200 mg bd (total 400 mg a day) vs cohort 1 placebo |
| (Archer *et al.*, 2017) | 30 | 29/30 (97) | 15 | 7/15 (47) | Cohort 2 Elagolix 300 mg bd (total 600 mg a day) vs cohort 2 placebo |
| (Archer *et al.*, 2017) | 31 | 26/31 (84) | 15 | 2/15 (13) | Cohort 4 Elagolix 400 mg qd (total 400 mg a day) vs cohort 4 placebo |
| (Archer *et al.*, 2017) | 28 | 26/28 (93) | 15 | 2/15 (13) | Elagolix 600 mg qd (total 600 mg a day) vs placebo |
| (Archer *et al.*, 2017) | 33 | 27/33 (83) | 33 | 28/33 (85) | Elagolix 200 mg bd (total 400 mg a day) vs Elagolix 200 mg bd + LD E2/NETA |
| (Archer *et al.*, 2017) | 30 | 29/30 (97) | 26 | 23/26 (88) | Elagolix 300 mg bd (total 600 mg a day) vs Elagolix 300 mg bd + CEP |
| (Carr *et al.*, 2018) | 62 | 57/62 (92) | 64 | 21/64 (33) | Elagolix 300 mg bd (total 600 mg a day) vs placebo |
| (Carr *et al.*, 2018) | 62 | 57/62 (92) | 61 | 54/61 (89) | Elagolix 300 mg bd (total 600 mg a day) vs Elagolix 300 mg bd + LD E2/NETA |
| (Carr *et al.*, 2018) | 62 | 57/62 (92) | 62 | 49/62 (79) | Elagolix 300 mg bd (total 600 mg a day) vs Elagolix 300 mg bd + SD E2/NETA |
| (Carr *et al.*, 2018) | 71 | 65/71 (92) | 76 | 28/76 (37) | Elagolix 600 mg qd (total 600 mg a day) vs placebo |
| (Carr *et al.*, 2018) | 71 | 65/71 (92) | 73 | 53/73 (73) | Elagolix 600 mg qd (total 600 mg a day) vs Elagolix 600 mg qd + LD E2/NETA |
| (Carr *et al.*, 2018) | 71 | 65/71 (92) | 76 | 65/76 (86) | Elagolix 600 mg qd (total 600 mg a day) vs Elagolix 600 mg qd + SD E2/NETA |
| *(Schlaff et al.*, 2020)  Elaris UF-1 | 104 | 87/104 (84) | 102 | 9/102 (9) | Elagolix 300 mg bd (total 600mg a day) vs placebo |
| *(Schlaff et al.*, 2020)  Elaris UF-1 | 104 | 87/104 (84) | 206 | 141/206 (68) | Elagolix 300 mg bd (total 600 mg a day) vs Elagolix 300 mg bd + SD E2/NETA |
| *(Schlaff et al.*, 2020)  Elaris UF-2 | 95 | 73/95 (77) | 94 | 9/94 (10) | Elagolix 300 mg bd (total 600mg a day) vs placebo |
| *(Schlaff et al.*, 2020)  Elaris UF-2 | 95 | 73/95 (77) | 189 | 145/189 (77) | Elagolix 300 mg bd (total 600 mg a day) vs Elagolix 300 mg bd + SD E2/NETA |
| *(Simon et al.*, 2020)  UF-EXTEND | 94 | 84/94 (89.4)  95% CI of percentage (83.1, 95.6) | 206 | 181/206 87.9)  95% CI of percentage (83.4, 92.3) | Elagolix 300 mg bd (total 600 mg a day) vs Elagolix 300 mg bd + SD E2/NETA |

| **Outcome: Reduction of >50% menstrual blood loss** | | | | | |
| --- | --- | --- | --- | --- | --- |
| **Author** | **Intervention group** | | **Control group** | | **Comments** |
|  | Number of participants analyzed | Percentage of participants  % | Number of participants analyzed | Percentage of participants  % |  |
| (Archer *et al.*, 2017) | 31 | 23/31 (74) | 15 | 2/15 (13) | Cohort 4 Elagolix 100 mg bd (total 200 mg a day) vs Cohort 4 placebo |
| (Archer *et al.*, 2017) | 33 | 30/33 (91) | 18 | 5/18 (28) | Cohort 1 Elagolix 200 mg bd (total 400 mg a day) vs cohort 1 placebo |
| (Archer *et al.*, 2017) | 30 | 29/30 (97) | 15 | 6/15 (40) | Cohort 2 Elagolix 300 mg bd (total 600 mg a day) vs cohort 2 placebo |
| (Archer *et al.*, 2017) | 31 | 26/31 (84) | 15 | 2/15 (13) | Cohort 4 Elagolix 400 mg qd ( total 400 mg a day) vs cohort 4 placebo |
| (Archer *et al.*, 2017) | 28 | 26/28 (93) | 15 | 2/15 (13) | Elagolix 600 mg qd (total 600 mg a day) vs placebo |
| (Archer *et al.*, 2017) | 33 | 30/33 (91) | 33 | 28/33 (85) | Elagolix 200 mg bd (total 400 mg a day) vs Elagolix 200 mg bd + LD E2/NETA |
| 67(Archer *et al.*, 2017) | 30 | 29/30 (97) | 26 | 23/26 (88) | Elagolix 300 mg bd (total 600 mg a day) vs Elagolix 300 mg bd + CEP |
| (Carr *et al.*, 2018) | 62 | 58/62 (94) | 64 | 20/64 (31) | Elagolix 300 mg bd (total 600 mg a day) vs placebo |
| (Carr *et al.*, 2018) | 62 | 58/62 (94) | 61 | 53/61 (87) | Elagolix 300 mg bd (total 600 mg a day) vs Elagolix 300 mg bd + LD E2/NETA |
| (Carr *et al.*, 2018) | 62 | 58/62 (94) | 62 | 51/62 (82) | Elagolix 300 mg bd (total 600 mg a day) vs Elagolix 300 mg bd + SD E2/NETA |
| (Carr *et al.*, 2018) | 71 | 64/71 (90) | 76 | 27/76 (36) | Elagolix 600 mg qd (total 600 mg a day) vs placebo |
| (Carr *et al.*, 2018) | 71 | 64/71 (90) | 73 | 58/73 (79) | Elagolix 600 mg qd (total 600 mg a day) vs Elagolix 600 mg qd + LD E2/NETA |
| (Carr *et al.*, 2018) | 71 | 64/71 (90) | 76 | 65/76 (86) | Elagolix 600 mg qd (total 600 mg a day) vs Elagolix 600 mg qd + SD E2/NETA |
| *(Schlaff et al.*, 2020)  Elaris UF-1 | 104 | 87/104 (84) | 102 | 9/102 (9) | Elagolix 300 mg bd (total 600mg a day) vs placebo |
| *(Schlaff et al.*, 2020)  Elaris UF-1 | 104 | 87/104 (84) | 206 | 141/206 (68) | Elagolix 300 mg bd (total 600 mg a day) vs Elagolix 300 mg bd + SD E2/NETA |
| *(Schlaff et al.*, 2020)  Elaris UF-2 | 95 | 73/95 (77) | 94 | 9/94 (10) | Elagolix 300 mg bd (total 600mg a day) vs placebo |
| *(Schlaff et al.*, 2020)  Elaris UF-2 | 95 | 73/95 (77) | 189 | 145/189 (77) | Elagolix 300 mg bd (total 600 mg a day) vs Elagolix 300 mg bd + SD E2/NETA |
| *(Simon et al.*, 2020)  UF-EXTEND | 94 | 84/94 (89.4)  95% CI of percentage (83.1, 95.6) | 206 | 181/206 87.9)  95% CI of percentage  (83.4, 92.3) | Elagolix 300 mg bd (total 600 mg a day) vs Elagolix 300 mg bd + SD E2/NETA |

| **Outcome: Reduction in uterine volume** | | | | | |
| --- | --- | --- | --- | --- | --- |
| **Author** | **Intervention group** | | **Control group** | | **Comments** |
|  | Number of participants analyzed | Percentage of participants  % | Number of participants analyzed | Percentage of participants  % |  |
| (Archer *et al.*, 2017) | 30 | 13/30 (43) | 15 | 1/15 (7) | Cohort 4 Elagolix 100 mg bd (total 200 mg a day) vs Cohort 4 placebo |
| (Archer *et al.*, 2017) | 31 | 15/31 (48) | 18 | 2/18 (11) | Cohort 1 Elagolix 200 mg bd (total 400 mg a day) vs cohort 1 placebo |
| (Archer *et al.*, 2017) | 29 | 20/29 (69) | 15 | 1/15 (7) | Cohort 2 Elagolix 300 mg bd (total 600 mg a day) vs cohort 2 placebo |
| (Archer *et al.*, 2017) | 30 | 16/30 (53) | 15 | 1/15 (7) | Cohort 4 Elagolix 400 mg qd (total 400 mg a day) vs cohort 4 placebo |
| (Archer *et al.*, 2017) | 27 | 15/27 (56) | 15 | 1/15 (7) | Elagolix 600 mg qd (total 600 mg a day) vs placebo |
| (Archer *et al.*, 2017) | 31 | 15/31 (48) | 33 | 14/33 (42) | Elagolix 200 mg bd (total 400 mg a day) vs Elagolix 200 mg bd + LD E2/NETA |
| (Archer *et al.*, 2017) | 29 | 20/29 (69) | 24 | 6/24 (25) | Elagolix 300 mg bd (total 600 mg a day) vs Elagolix 300 mg bd + CEP |

| **Outcome: Hemoglobin Level** | | | | | |
| --- | --- | --- | --- | --- | --- |
| **Author** | **Intervention group** | | **Control group** | | **Comments** |
|  | Number of participants analyzed | Percentage of participants  % | Number of participants analyzed | Number of participants  % |  |
| (Archer *et al.*, 2017) | 24 | 17/24 (71) | 11 | 1/11 (9) | Cohort 4 Elagolix 100 mg bd (total 200 mg a day) vs Cohort 4 placebo |
| (Archer *et al.*, 2017) | 27 | 16/27 (59) | 14 | 4/14 (29) | Cohort 1 Elagolix 200 mg bd (total 400 mg a day) vs cohort 1 placebo |
| (Archer *et al.*, 2017) | 25 | 13/25 (52) | 14 | 4/14 (29) | Cohort 2 Elagolix 300 mg bd (total 600 mg a day) vs cohort 2 placebo |
| (Archer *et al.*, 2017) | 23 | 14/23 (61) | 11 | 1/11 (9) | Cohort 4 Elagolix 400 mg qd ( total 400 mg a day) vs cohort 4 placebo |
| (Archer *et al.*, 2017) | 23 | 13/23 (57) | 11 | 1/11 (9) | Elagolix 600 mg qd ( total 600 mg a day) vs placebo |
| (Archer *et al.*, 2017) | 27 | 16/27 (59) | 28 | 12/28 (43) | Elagolix 200 mg bd (total 400 mg a day) vs Elagolix 200 mg bd + LD E2/NETA |
| (Archer *et al.*, 2017) | 25 | 13/25 (52) | 21 | 13/21 (62) | Elagolix 300 mg bd (total 600 mg a day) vs Elagolix 300 mg bd + CEP |
| (Carr *et al.*, 2018) | 61 | 49/61 (80) | 64 | 19/64 (30) | Elagolix 300 mg bd (total 600 mg a day) vs placebo |
| (Carr *et al.*, 2018) | 61 | 49/61 (80) | 59 | 41/59 (70) | Elagolix 300 mg bd (total 600 mg a day) vs Elagolix 300 mg bd + LD E2/NETA |
| (Carr *et al.*, 2018) | 61 | 49/61 (80) | 61 | 36/61 (59) | Elagolix 300 mg bd (total 600 mg a day) vs Elagolix 300 mg bd + SD E2/NETA |
| (Carr *et al.*, 2018) | 71 | 37/71 (52) | 76 | 20/76 (26) | Elagolix 600 mg qd (total 600 mg a day) vs placebo |
| (Carr *et al.*, 2018) | 71 | 37/71 (52) | 72 | 37/72 (51) | Elagolix 600 mg qd (total 600 mg a day) vs Elagolix 600 mg qd + LD E2/NETA |
| (Carr *et al.*, 2018) | 71 | 37/71 (52) | 74 | 39/74 (53) | Elagolix 600 mg qd (total 600 mg a day) vs Elagolix 600 mg qd + SD E2/NETA |
| *(Schlaff et al.*, 2020)  Elaris UF-1 | 41 | 27/41 (66) | 31 | 5/31 (16) | Elagolix 300 mg bd (total 600mg a day) vs placebo |
| *(Schlaff et al.*, 2020)  Elaris UF-1 | 41 | 27/41 (66) | 52 | 32/52 (62) | Elagolix 300 mg bd (total 600 mg a day) vs Elagolix 300 mg bd + SD E2/NETA |
| *(Schlaff et al.*, 2020)  Elaris UF-2 | 25 | 10/25 (40) | 24 | 5/24 (21) | Elagolix 300 mg bd (total 600mg a day) vs placebo |
| *(Schlaff et al.*, 2020)  Elaris UF-2 | 25 | 10/25 (40) | 28 | 24/28 (50) | Elagolix 300 mg bd (total 600 mg a day) vs Elagolix 300 mg bd + SD E2/NETA |
| *(Simon et al.*, 2020)  UF-EXTEND | 94 | 67/94(71.4)  95% CI of percentage (54.7, 88.2) | 206 | 149/206 (72.5)  95% CI of percentage (60.3, 84.8) | Elagolix 300 mg bd (total 600 mg a day) vs Elagolix 300 mg bd + SD E2/NETA |

| **Outcome: Adverse Event (Any AE)** | | | | | |
| --- | --- | --- | --- | --- | --- |
| **Author** | **Intervention group** | | **Control group** | | **Comments** |
|  | Number of participants analyzed | Percentage of participants  (%) | Number of participants analyzed | Percentage of participants  (%) |  |
| (Archer *et al.*, 2017) | 33 | 24/33 (72.7) | 16 | 9/16 (56.2) | Cohort 4 Elagolix 100 mg bd (total 200 mg a day) vs Cohort 4 placebo |
| (Archer *et al.*, 2017) | 35 | 28/35 (80) | 18 | 8/18 (44.4) | Cohort 1 Elagolix 200 mg bd (total 400 mg a day) vs cohort 1 placebo |
| (Archer *et al.*, 2017) | 30 | 21/30 (70) | 16 | 10/16 (62.5) | Cohort 2 Elagolix 300 mg bd (total 600 mg a day) vs cohort 2 placebo |
| (Archer *et al.*, 2017) | 32 | 26/32 (81.3 | 16 | 9/16 (56.2) | Cohort 4 Elagolix 400 mg qd ( total 400 mg a day) vs cohort 4 placebo |
| (Archer *et al.*, 2017) | 30 | 22/30 (73.3) | 16 | 9/16 (56.2) | Elagolix 600 mg qd ( total 600 mg a day) vs placebo |
| (Archer *et al.*, 2017) | 35 | 28/35 (80) | 34 | 24/34 (70.6) | Elagolix 200 mg bd (total 400 mg a day) vs Elagolix 200 mg bd + LD E2/NETA |
| (Archer *et al.*, 2017) | 30 | 21/30 (70) | 27 | 15/27 (55.6) | Elagolix 300 mg bd (total 600 mg a day) vs Elagolix 300 mg bd + CEP |
| (Carr *et al.*, 2018) | 65 | 52/65 (80) | 65 | 47/65 (72) | Elagolix 300 mg bd (total 600 mg a day) vs placebo |
| (Carr *et al.*, 2018) | 65 | 52/65 (80) | 64 | 47/64 (73) | Elagolix 300 mg bd (total 600 mg a day) vs Elagolix 300 mg bd + LD E2/NETA |
| (Carr *et al.*, 2018) | 65 | 52/65 (80) | 65 | 48/65 (74) | Elagolix 300 mg bd (total 600 mg a day) vs Elagolix 300 mg bd + SD E2/NETA |
| (Carr *et al.*, 2018) | 77 | 67/77 (87) | 78 | 53/78 (68) | Elagolix 600 mg qd (total 600 mg a day) vs placebo |
| (Carr *et al.*, 2018) | 77 | 67/77 (87) | 76 | 56/76 (74) | Elagolix 600 mg qd (total 600 mg a day) vs Elagolix 600 mg qd + LD E2/NETA |
| (Carr *et al.*, 2018) | 77 | 67/77 (87) | 77 | 55/77 (71) | Elagolix 600 mg qd (total 600 mg a day) vs Elagolix 600 mg qd + SD E2/NETA |
| *(Schlaff et al.*, 2020)  Elaris UF-1 | 104 | 94/104 (90.4) | 102 | 71/102 (69.6) | Elagolix 300 mg bd (total 600mg a day) vs placebo |
| *(Schlaff et al.*, 2020)  Elaris UF-1 | 104 | 94/104 (90.4) | 206 | 140/206 (68) | Elagolix 300 mg bd (total 600 mg a day) vs Elagolix 300 mg bd + SD E2/NETA |
| *(Schlaff et al.*, 2020)  Elaris UF-2 | 95 | 72/95 (76) | 94 | 59/94 (63) | Elagolix 300 mg bd (total 600mg a day) vs placebo |
| *(Schlaff et al.*, 2020)  Elaris UF-2 | 95 | 72/95 (76) | 189 | 143/189 (75,7 | Elagolix 300 mg bd (total 600 mg a day) vs Elagolix 300 mg bd + SD E2/NETA |
| *(Simon et al.*, 2020)  UF-EXTEND | 98 | 90/98 (92) | 218 | 186/218 (85) | Elagolix 300 mg bd (total 600 mg a day) vs Elagolix 300 mg bd + SD E2/NETA |

| **Outcome: Adverse Event (Serious AE)** | | | | | |
| --- | --- | --- | --- | --- | --- |
| **Author** | **Intervention group** | | **Control group** | | **Comments** |
|  | Number of participants analyzed | Percentage of participants  (%) | Number of participants analyzed | Percentage of participants  (%) |  |
| (Archer *et al.*, 2017) | 33 | 2/33 (6.1) | 16 | 2/16 )12.5) | Cohort 4 Elagolix 100 mg bd (total 200 mg a day) vs Cohort 4 placebo |
| (Archer *et al.*, 2017) | 35 | 0 | 18 | 1/18 (5.5) | Cohort 1 Elagolix 200 mg bd (total 400 mg a day) vs cohort 1 placebo |
| (Archer *et al.*, 2017) | 30 | 1/30 (3.3) | 16 | 0 | Cohort 2 Elagolix 300 mg bd (total 600 mg a day) vs cohort 2 placebo |
| (Archer *et al.*, 2017) | 32 | 0 | 16 | 2/16 )12.5) | Cohort 4 Elagolix 400 mg qd ( total 400 mg a day) vs cohort 4 placebo |
| (Archer *et al.*, 2017) | 30 | 2/30 (6.7) | 16 | 2/16 )12.5) | Elagolix 600 mg qd ( total 600 mg a day) vs placebo |
| (Archer *et al.*, 2017) | 35 | 0 | 34 | 0 | Elagolix 200 mg bd (total 400 mg a day) vs Elagolix 200 mg bd + LD E2/NETA |
| (Archer *et al.*, 2017) | 30 | 1/30 (3.3) | 27 | 0 | Elagolix 300 mg bd (total 600 mg a day) vs Elagolix 300 mg bd + CEP |
| (Carr *et al.*, 2018) | 65 | 3/65 (4.6) | 65 | 6/65 (9.2) | Elagolix 300 mg bd (total 600 mg a day) vs placebo |
| (Carr *et al.*, 2018) | 65 | 3/65 (4.6) | 64 | 3/64 (4.7) | Elagolix 300 mg bd (total 600 mg a day) vs Elagolix 300 mg bd + LD E2/NETA |
| (Carr *et al.*, 2018) | 65 | 3/65 (4.6) | 65 | 1/65 (1.5) | Elagolix 300 mg bd (total 600 mg a day) vs Elagolix 300 mg bd + SD E2/NETA |
| (Carr *et al.*, 2018) | 77 | 5/77 (6.5) | 78 | 1/78 (1.3) | Elagolix 600 mg qd (total 600 mg a day) vs placebo |
| (Carr *et al.*, 2018) | 77 | 5/77 (6.5) | 76 | 3/76 (3.9) | Elagolix 600 mg qd (total 600 mg a day) vs Elagolix 600 mg qd + LD E2/NETA |
| (Carr *et al.*, 2018) | 77 | 5/77 (6.5) | 77 | 4/77 (5.2) | Elagolix 600 mg qd (total 600 mg a day) vs Elagolix 600 mg qd + SD E2/NETA |
| *(Schlaff et al.*, 2020)  Elaris UF-1 | 104 | 3/104 (2.9) | 102 | 5/102 (4.9) | Elagolix 300 mg bd (total 600mg a day) vs placebo |
| *(Schlaff et al.*, 2020)  Elaris UF-1 | 104 | 3/104 (2.9) | 206 | 3/206 (1.5) | Elagolix 300 mg bd (total 600 mg a day) vs Elagolix 300 mg bd + SD E2/NETA |
| *(Schlaff et al.*, 2020)  Elaris UF-2 | 95 | 4/95 (4) | 94 | 1/94 (1) | Elagolix 300 mg bd (total 600mg a day) vs placebo |
| *(Schlaff et al.*, 2020)  Elaris UF-2 | 95 | 4/95 (4) | 189 | 7/189 (3.7) | Elagolix 300 mg bd (total 600 mg a day) vs Elagolix 300 mg bd + SD E2/NETA |
| *(Simon et al.*, 2020)  UF-EXTEND | 98 | 2/98 (2) | 218 | 8/218 (4) | Elagolix 300 mg bd (total 600 mg a day) vs Elagolix 300 mg bd + SD E2/NETA |

| **Outcome: Adverse Event (Severe AE)** | | | | | |
| --- | --- | --- | --- | --- | --- |
| **Author** | **Intervention group** | | **Control group** | | **Comments** |
|  | Number of participants analyzed | Percentage of participants  (%) | Number of participants analyzed | Percentage of participants  (%) |  |
| (Archer *et al.*, 2017) | 33 | 3/33 (9.1) | 16 | 2/16 (12.5) | Cohort 4 Elagolix 100 mg bd (total 200 mg a day) vs Cohort 4 placebo |
| (Archer *et al.*, 2017) | 35 | 3/35 (8.6) | 18 | 1/18 (5.5) | Cohort 1 Elagolix 200 mg bd (total 400 mg a day) vs cohort 1 placebo |
| (Archer *et al.*, 2017) | 30 | 2/30 (6.7) | 16 | 2/16 (12.5) | Cohort 2 Elagolix 300 mg bd (total 600 mg a day) vs cohort 2 placebo |
| (Archer *et al.*, 2017) | 32 | 5/32 (15.6) | 16 | 2/16 (12.5) | Cohort 4 Elagolix 400 mg qd ( total 400 mg a day) vs cohort 4 placebo |
| (Archer *et al.*, 2017) | 30 | 3/30 (10) | 16 | 2/16 (12.5) | Elagolix 600 mg qd ( total 600 mg a day) vs placebo |
| (Archer *et al.*, 2017) | 35 | 3/35 (8.6) | 34 | 0 | Elagolix 200 mg bd (total 400 mg a day) vs Elagolix 200 mg bd + LD E2/NETA |
| (Archer *et al.*, 2017) | 30 | 2/30 (6.7) | 27 | 3/27 (11.1) | Elagolix 300 mg bd (total 600 mg a day) vs Elagolix 300 mg bd + CEP |
| (Carr *et al.*, 2018) | 65 | * | 65 | * | Elagolix 300 mg bd (total 600 mg a day) vs placebo |
| (Carr *et al.*, 2018) | 65 | * | 64 | * | Elagolix 300 mg bd (total 600 mg a day) vs Elagolix 300 mg bd + LD E2/NETA |
| (Carr *et al.*, 2018) | 65 | * | 65 | * | Elagolix 300 mg bd (total 600 mg a day) vs Elagolix 300 mg bd + SD E2/NETA |
| (Carr *et al.*, 2018) | 77 | * | 78 | * | Elagolix 600 mg qd (total 600 mg a day) vs placebo |
| (Carr *et al.*, 2018) | 77 | * | 76 | * | Elagolix 600 mg qd (total 600 mg a day) vs Elagolix 600 mg qd + LD E2/NETA |
| (Carr *et al.*, 2018) | 77 | * | 77 | * | Elagolix 600 mg qd (total 600 mg a day) vs Elagolix 600 mg qd + SD E2/NETA |
| *(Schlaff et al.*, 2020)  Elaris UF-1 | 104 | 9/104 (8.7) | 102 | 4/102 (3.9) | Elagolix 300 mg bd (total 600mg a day) vs placebo |
| *(Schlaff et al.*, 2020)  Elaris UF-1 | 104 | 9/104 (8.7) | 206 | 19/206 (9.2) | Elagolix 300 mg bd (total 600 mg a day) vs Elagolix 300 mg bd + SD E2/NETA |
| *(Schlaff et al.*, 2020)  Elaris UF-2 | 95 | 11/95 (12) | 94 | 6/94 (6) | Elagolix 300 mg bd (total 600mg a day) vs placebo |
| *(Schlaff et al.*, 2020)  Elaris UF-2 | 95 | 11/95 (12) | 189 | 17/189 (9.0) | Elagolix 300 mg bd (total 600 mg a day) vs Elagolix 300 mg bd + SD E2/NETA |
| *(Simon et al.*, 2020)  UF-EXTEND | 98 | 5/98 (5) | 218 | 29/218 (13) | Elagolix 300 mg bd (total 600 mg a day) vs Elagolix 300 mg bd + SD E2/NETA |

***** Data not reported in article

| **Outcome: Adverse Event (AE led to discontinuation)** | | | | | |
| --- | --- | --- | --- | --- | --- |
| **Author** | **Intervention group** | | **Control group** | | **Comments** |
|  | Number of participants analyzed | Percentage of participants  (%) | Number of participants analyzed | Percentage of participants  (%) |  |
| (Archer *et al.*, 2017) | 33 | 4/33 (12.1) | 16 | 2/16 (12.5) | Cohort 4 Elagolix 100 mg bd (total 200 mg a day) vs Cohort 4 placebo |
| (Archer *et al.*, 2017) | 35 | 5/35 (14.3) | 18 | 1/18 (5.5) | Cohort 1 Elagolix 200 mg bd (total 400 mg a day) vs cohort 1 placebo |
| (Archer *et al.*, 2017) | 30 | 2/30 (6.7) | 16 | 1/16 (6.25) | Cohort 2 Elagolix 300 mg bd (total 600 mg a day) vs cohort 2 placebo |
| (Archer *et al.*, 2017) | 32 | 4/32 (12.5) | 16 | 2/16 (12.5) | Cohort 4 Elagolix 400 mg qd (total 400 mg a day) vs cohort 4 placebo |
| (Archer *et al.*, 2017) | 30 | 3/30 (10) | 16 | 2/16 (12.5) | Elagolix 600 mg qd ( total 600 mg a day) vs placebo |
| (Archer *et al.*, 2017) | 35 | 5/35 (14.3) | 34 | 2/34 (5.9) | Elagolix 200 mg bd (total 400 mg a day) vs Elagolix 200 mg bd + LD E2/NETA |
| (Archer *et al.*, 2017) | 30 | 2/30 (6.7) | 27 | 0 | Elagolix 300 mg bd (total 600 mg a day) vs Elagolix 300 mg bd + CEP |
| (Carr *et al.*, 2018) | 65 | 4/65 (6.2) | 65 | 7/65 (11) | Elagolix 300 mg bd (total 600 mg a day) vs placebo |
| (Carr *et al.*, 2018) | 65 | 4/65 (6.2) | 64 | 2/64 (3.1) | Elagolix 300 mg bd (total 600 mg a day) vs Elagolix 300 mg bd + LD E2/NETA |
| (Carr *et al.*, 2018) | 65 | 4/65 (6.2) | 65 | 6/65 (9.2) | Elagolix 300 mg bd (total 600 mg a day) vs Elagolix 300 mg bd + SD E2/NETA |
| (Carr *et al.*, 2018) | 77 | 11/77 (14) | 78 | 1/78 (1.3) | Elagolix 600 mg qd (total 600 mg a day) vs placebo |
| (Carr *et al.*, 2018) | 77 | 11/77 (14) | 76 | 7/76 (9.2) | Elagolix 600 mg qd (total 600 mg a day) vs Elagolix 600 mg qd + LD E2/NETA |
| (Carr *et al.*, 2018) | 77 | 11/77 (14) | 77 | 9/77 (12) | Elagolix 600 mg qd (total 600 mg a day) vs Elagolix 600 mg qd + SD E2/NETA |
| *(Schlaff et al.*, 2020)  Elaris UF-1 | 104 | 10/104 (9.6) | 102 | 8/102 (7.8) | Elagolix 300 mg bd (total 600mg a day) vs placebo |
| *(Schlaff et al.*, 2020)  Elaris UF-1 | 104 | 10/104 (9.6) | 206 | 22/206 (10.7) | Elagolix 300 mg bd (total 600 mg a day) vs Elagolix 300 mg bd + SD E2/NETA |
| *(Schlaff et al.*, 2020)  Elaris UF-2 | 95 | 12/95 (13) | 94 | 5/94 (5) | Elagolix 300 mg bd (total 600mg a day) vs placebo |
| *(Schlaff et al.*, 2020)  Elaris UF-2 | 95 | 12/95 (13) | 189 | 16/189 (8.5) | Elagolix 300 mg bd (total 600 mg a day) vs Elagolix 300 mg bd + SD E2/NETA |
| *(Simon et al.*, 2020)  UF-EXTEND | 98 | 7/98 (7) | 218 | 8/218 (4) | Elagolix 300 mg bd (total 600 mg a day) vs Elagolix 300 mg bd + SD E2/NETA |

| **Outcome: Adverse Event (Hot flush)** | | | | | |
| --- | --- | --- | --- | --- | --- |
| **Author** | **Intervention group** | | **Control group** | | **Comments** |
|  | Number of participants analyzed | Percentage of participants  (%) | Number of participants analyzed | Percentage of participants  (%) |  |
| (Archer *et al.*, 2017) | 33 | 15/33 (45.5) | 16 | 2/16 (12.5) | Cohort 4 Elagolix 100 mg bd (total 200 mg a day) vs Cohort 4 placebo |
| (Archer *et al.*, 2017) | 35 | 19/35 (54.3) | 18 | 1/18 (5.5) | Cohort 1 Elagolix 200 mg bd (total 400 mg a day) vs cohort 1 placebo |
| (Archer *et al.*, 2017) | 30 | 15/30 (50) | 16 | 3/16 (18.7) | Cohort 2 Elagolix 300 mg bd (total 600 mg a day) vs cohort 2 placebo |
| (Archer *et al.*, 2017) | 32 | 20/32 (62.5) | 16 | 2/16 (12.5) | Cohort 4 Elagolix 400 mg qd (total 400 mg a day) vs cohort 4 placebo |
| (Archer *et al.*, 2017) | 30 | 15/30 (50) | 16 | 2/16 (12.5) | Elagolix 600 mg qd ( total 600 mg a day) vs placebo |
| (Archer *et al.*, 2017) | 35 | 19/35 (54.3) | 34 | 9/34 (26.5) | Elagolix 200 mg bd (total 400 mg a day) vs Elagolix 200 mg bd + LD E2/NETA |
| (Archer *et al.*, 2017) | 30 | 15/30 (50) | 27 | 5/27 (18.5) | Elagolix 300 mg bd (total 600 mg a day) vs Elagolix 300 mg bd + CEP |
| (Carr *et al.*, 2018) | 65 | 29/65 (45) | 65 | 2/65 (3.1) | Elagolix 300 mg bd (total 600 mg a day) vs placebo |
| (Carr *et al.*, 2018) | 65 | 29/65 (45) | 64 | 16/64 (25) | Elagolix 300 mg bd (total 600 mg a day) vs Elagolix 300 mg bd + LD E2/NETA |
| (Carr *et al.*, 2018) | 65 | 29/65 (45) | 65 | 7/65 (11) | Elagolix 300 mg bd (total 600 mg a day) vs Elagolix 300 mg bd + SD E2/NETA |
| (Carr *et al.*, 2018) | 77 | 38/77 (49) | 78 | 4/78 (5.1) | Elagolix 600 mg qd (total 600 mg a day) vs placebo |
| (Carr *et al.*, 2018) | 77 | 38/77 (49) | 76 | 14/76 (18) | Elagolix 600 mg qd (total 600 mg a day) vs Elagolix 600 mg qd + LD E2/NETA |
| (Carr *et al.*, 2018) | 77 | 38/77 (49) | 77 | 11/77 (14) | Elagolix 600 mg qd (total 600 mg a day) vs Elagolix 600 mg qd + SD E2/NETA |
| *(Schlaff et al.*, 2020)  Elaris UF-1 | 104 | 67/104 (64.4) | 102 | 9/102 (8.8) | Elagolix 300 mg bd (total 600mg a day) vs placebo |
| *(Schlaff et al.*, 2020)  Elaris UF-1 | 104 | 67/104 (64.4) | 206 | 42/206 (20.4) | Elagolix 300 mg bd (total 600 mg a day) vs Elagolix 300 mg bd + SD E2/NETA |
| *(Schlaff et al.*, 2020)  Elaris UF-2 | 95 | 41/95 (43) | 94 | 4/94 (4) | Elagolix 300 mg bd (total 600mg a day) vs placebo |
| *(Schlaff et al.*, 2020)  Elaris UF-2 | 95 | 41/95 (43) | 189 | 37/189 (19.6) | Elagolix 300 mg bd (total 600 mg a day) vs Elagolix 300 mg bd + SD E2/NETA |
| *(Simon et al.*, 2020)  UF-EXTEND | 98 | 64/98 (65) | 218 | 54/218 (25) | Elagolix 300 mg bd (total 600 mg a day) vs Elagolix 300 mg bd + SD E2/NETA |

| **Outcome: Adverse Event (Headache)** | | | | | |
| --- | --- | --- | --- | --- | --- |
| **Author** | **Intervention group** | | **Control group** | | **Comments** |
|  | Number of participants analyzed | Percentage of participants  (%) | Number of participants analyzed | Percentage of participants  (%) |  |
| (Archer *et al.*, 2017) | 33 | 3/33 (9.1) | 16 | 0 | Cohort 4 Elagolix 100 mg bd (total 200 mg a day) vs Cohort 4 placebo |
| (Archer *et al.*, 2017) | 35 | 3/35 (8.6) | 18 | 0 | Cohort 1 Elagolix 200 mg bd (total 400 mg a day) vs cohort 1 placebo |
| (Archer *et al.*, 2017) | 30 | 6/30 (20) | 16 | 3/16 (18.7) | Cohort 2 Elagolix 300 mg bd (total 600 mg a day) vs cohort 2 placebo |
| (Archer *et al.*, 2017) | 32 | 4/32 (12.5) | 16 | 0 | Cohort 4 Elagolix 400 mg qd (total 400 mg a day) vs cohort 4 placebo |
| (Archer *et al.*, 2017) | 30 | 9/30 (30) | 16 | 0 | Elagolix 600 mg qd ( total 600 mg a day) vs placebo |
| (Archer *et al.*, 2017) | 35 | 3/35 (8.6) | 34 | 5/34 (14.7) | Elagolix 200 mg bd (total 400 mg a day) vs Elagolix 200 mg bd + LD E2/NETA |
| (Archer *et al.*, 2017) | 30 | 6/30 (20) | 27 | 2/27 (7.4 | Elagolix 300 mg bd (total 600 mg a day) vs Elagolix 300 mg bd + CEP |
| (Carr *et al.*, 2018) | 65 | 8/65 (12) | 65 | 6/65 (9.2) | Elagolix 300 mg bd (total 600 mg a day) vs placebo |
| (Carr *et al.*, 2018) | 65 | 8/65 (12) | 64 | 9/64 (14) | Elagolix 300 mg bd (total 600 mg a day) vs Elagolix 300 mg bd + LD E2/NETA |
| (Carr *et al.*, 2018) | 65 | 8/65 (12) | 65 | 13/65 (20) | Elagolix 300 mg bd (total 600 mg a day) vs Elagolix 300 mg bd + SD E2/NETA |
| (Carr *et al.*, 2018) | 77 | 13/77(17) | 78 | 8/78 (10) | Elagolix 600 mg qd (total 600 mg a day) vs placebo |
| (Carr *et al.*, 2018) | 77 | 13/77(17) | 76 | 11/76 (15) | Elagolix 600 mg qd (total 600 mg a day) vs Elagolix 600 mg qd + LD E2/NETA |
| (Carr *et al.*, 2018) | 77 | 13/77(17) | 77 | 14/77 (18) | Elagolix 600 mg qd (total 600 mg a day) vs Elagolix 600 mg qd + SD E2/NETA |
| *(Schlaff et al.*, 2020)  Elaris UF-1 | 104 | 17/104 (6.3) | 102 | 9/102 (8.8) | Elagolix 300 mg bd (total 600mg a day) vs placebo |
| *(Schlaff et al.*, 2020)  Elaris UF-1 | 104 | 17/104 (6.3) | 206 | 17/206 (8.3) | Elagolix 300 mg bd (total 600 mg a day) vs Elagolix 300 mg bd + SD E2/NETA |
| *(Schlaff et al.*, 2020)  Elaris UF-2 | 95 | 13/95 (14) | 94 | 5/94 (5) | Elagolix 300 mg bd (total 600mg a day) vs placebo |
| *(Schlaff et al.*, 2020)  Elaris UF-2 | 95 | 13/95 (14) | 189 | 20/189 (10.6) | Elagolix 300 mg bd (total 600 mg a day) vs Elagolix 300 mg bd + SD E2/NETA |
| *(Simon et al.*, 2020)  UF-EXTEND | 98 | 13/98 (13) | 218 | 26/218 (12) | Elagolix 300 mg bd (total 600 mg a day) vs Elagolix 300 mg bd + SD E2/NETA |

| **Outcome: Adverse Event (Abdominal pain)** | | | | | |
| --- | --- | --- | --- | --- | --- |
| **Author** | **Intervention group** | | **Control group** | | **Comments** |
|  | Number of participants analyzed | Percentage of participants  (%) | Number of participants analyzed | Percentage of participants  (%) |  |
| (Archer *et al.*, 2017) | 33 | 1/33 (3.0) | 16 | 0 | Cohort 4 Elagolix 100 mg bd (total 200 mg a day) vs Cohort 4 placebo |
| (Archer *et al.*, 2017) | 35 | 2/35 (5.7) | 18 | 1/18 (5.5) | Cohort 1 Elagolix 200 mg bd (total 400 mg a day) vs cohort 1 placebo |
| (Archer *et al.*, 2017) | 30 | 3/30 (10) | 16 | 0 | Cohort 2 Elagolix 300 mg bd (total 600 mg a day) vs cohort 2 placebo |
| (Archer *et al.*, 2017) | 32 | 1/32 (3.1) | 16 | 0 | Cohort 4 Elagolix 400 mg qd (total 400 mg a day) vs cohort 4 placebo |
| (Archer *et al.*, 2017) | 30 | 2/30 (6.7) | 16 | 0 | Elagolix 600 mg qd ( total 600 mg a day) vs placebo |
| (Archer *et al.*, 2017) | 35 | 2/35 (5.7) | 34 | 0 | Elagolix 200 mg bd (total 400 mg a day) vs Elagolix 200 mg bd + LD E2/NETA |
| (Archer *et al.*, 2017) | 30 | 3/30 (10) | 27 | 4/27 (14.8) | Elagolix 300 mg bd (total 600 mg a day) vs Elagolix 300 mg bd + CEP |
| (Carr *et al.*, 2018) | 65 | 1/65 (1.5) | 65 | 2/65 (3.1) | Elagolix 300 mg bd (total 600 mg a day) vs placebo |
| (Carr *et al.*, 2018) | 65 | 1/65 (1.5) | 64 | 2/64 (3.1) | Elagolix 300 mg bd (total 600 mg a day) vs Elagolix 300 mg bd + LD E2/NETA |
| (Carr *et al.*, 2018) | 65 | 1/65 (1.5) | 65 | 6/65 (9.2) | Elagolix 300 mg bd (total 600 mg a day) vs Elagolix 300 mg bd + SD E2/NETA |
| (Carr *et al.*, 2018) | 77 | 3/77 (3.9) | 78 | 3/78 (3.8) | Elagolix 600 mg qd (total 600 mg a day) vs placebo |
| (Carr *et al.*, 2018) | 77 | 3/77 (3.9) | 76 | 3/76 (3.9) | Elagolix 600 mg qd (total 600 mg a day) vs Elagolix 600 mg qd + LD E2/NETA |
| (Carr *et al.*, 2018) | 77 | 3/77 (3.9) | 77 | 4/77 (5.2) | Elagolix 600 mg qd (total 600 mg a day) vs Elagolix 600 mg qd + SD E2/NETA |

| **Outcome: Adverse Event (Dizziness)** | | | | | |
| --- | --- | --- | --- | --- | --- |
| **Author** | **Intervention group** | | **Control group** | | **Comments** |
|  | Number of participants analyzed | Percentage of participants  (%) | Number of participants analyzed | Percentage of participants  (%) |  |
| (Archer *et al.*, 2017) | 33 | 1/33 (3.0) | 16 | 0 | Cohort 4 Elagolix 100 mg bd (total 200 mg a day) vs Cohort 4 placebo |
| (Archer *et al.*, 2017) | 35 | 2/35 (5.7) | 18 | 0 | Cohort 1 Elagolix 200 mg bd (total 400 mg a day) vs cohort 1 placebo |
| (Archer *et al.*, 2017) | 30 | 3/30 (10) | 16 | 2/16 (12.2) | Cohort 2 Elagolix 300 mg bd (total 600 mg a day) vs cohort 2 placebo |
| (Archer *et al.*, 2017) | 32 | 3/32 (9.4) | 16 | 0 | Cohort 4 Elagolix 400 mg qd (total 400 mg a day) vs cohort 4 placebo |
| (Archer *et al.*, 2017) | 30 | 6/30 (20) | 16 | 0 | Elagolix 600 mg qd (total 600 mg a day) vs placebo |
| (Archer *et al.*, 2017) | 35 | 2/35 (5.7) | 34 | 3/34 (8.8) | Elagolix 200 mg bd (total 400 mg a day) vs Elagolix 200 mg bd + LD E2/NETA |
| (Archer *et al.*, 2017) | 30 | 3/30 (10) | 27 | 1/27 (3.7) | Elagolix 300 mg bd (total 600 mg a day) vs Elagolix 300 mg bd + CEP |
| (Carr *et al.*, 2018) | 65 | 3/65 (4.6) | 65 | 3/65 (4.6) | Elagolix 300 mg bd (total 600 mg a day) vs placebo |
| (Carr *et al.*, 2018) | 65 | 3/65 (4.6) | 64 | 1/64 (1.15) | Elagolix 300 mg bd (total 600 mg a day) vs Elagolix 300 mg bd + LD E2/NETA |
| (Carr *et al.*, 2018) | 65 | 3/65 (4.6) | 65 | 3/65 (4.6) | Elagolix 300 mg bd (total 600 mg a day) vs Elagolix 300 mg bd + SD E2/NETA |
| (Carr *et al.*, 2018) | 77 | 3/77 (3.9) | 78 | 4/78 (5.1) | Elagolix 600 mg qd (total 600 mg a day) vs placebo |
| (Carr *et al.*, 2018) | 77 | 3/77 (3.9) | 76 | 5/76 (6.5) | Elagolix 600 mg qd (total 600 mg a day) vs Elagolix 600 mg qd + LD E2/NETA |
| (Carr *et al.*, 2018) | 77 | 3/77 (3.9) | 77 | 5/77 (6.5) | Elagolix 600 mg qd (total 600 mg a day) vs Elagolix 600 mg qd + SD E2/NETA |

| **Outcome: Adverse Event (Nausea)** | | | | | |
| --- | --- | --- | --- | --- | --- |
| **Author** | **Intervention group** | | **Control group** | | **Comments** |
|  | Number of participants analyzed | Percentage of participants  (%) | Number of participants analyzed | Percentage of participants  (%) |  |
| (Archer *et al.*, 2017) | 33 | 0 | 16 | 1/16 (6.25) | Cohort 4 Elagolix 100 mg bd (total 200 mg a day) vs Cohort 4 placebo |
| (Archer *et al.*, 2017) | 35 | 3/35 (8.6) | 18 | 0 | Cohort 1 Elagolix 200 mg bd (total 400 mg a day) vs cohort 1 placebo |
| (Archer *et al.*, 2017) | 30 | 2/30 (6.7) | 16 | 2/16 (12.2) | Cohort 2 Elagolix 300 mg bd (total 600 mg a day) vs cohort 2 placebo |
| (Archer *et al.*, 2017) | 32 | 5/32 (15.6) | 16 | 1/16 (6.25) | Cohort 4 Elagolix 400 mg qd (total 400 mg a day) vs cohort 4 placebo |
| (Archer *et al.*, 2017) | 30 | 9/30 (30) | 16 | 1/16 (6.25) | Elagolix 600 mg qd ( total 600 mg a day) vs placebo |
| (Archer *et al.*, 2017) | 35 | 3/35 (8.6) | 34 | 1/34 (2.9) | Elagolix 200 mg bd (total 400 mg a day) vs Elagolix 200 mg bd + LD E2/NETA |
| (Archer *et al.*, 2017) | 30 | 2/30 (6.7) | 27 | 4/27 (14.8) | Elagolix 300 mg bd (total 600 mg a day) vs Elagolix 300 mg bd + CEP |
| (Carr *et al.*, 2018) | 65 | 4/65 (6.2) | 65 | 6/65 (9.2) | Elagolix 300 mg bd (total 600 mg a day) vs placebo |
| (Carr *et al.*, 2018) | 65 | 4/65 (6.2) | 64 | 4/64 (6.3) | Elagolix 300 mg bd (total 600 mg a day) vs Elagolix 300 mg bd + LD E2/NETA |
| (Carr *et al.*, 2018) | 65 | 4/65 (6.2) | 65 | 12/65 (19) | Elagolix 300 mg bd (total 600 mg a day) vs Elagolix 300 mg bd + SD E2/NETA |
| (Carr *et al.*, 2018) | 77 | 10/77 (13) | 78 | 3/78 (2.8) | Elagolix 600 mg qd (total 600 mg a day) vs placebo |
| (Carr *et al.*, 2018) | 77 | 10/77 (13) | 76 | 12/76 (16) | Elagolix 600 mg qd (total 600 mg a day) vs Elagolix 600 mg qd + LD E2/NETA |
| (Carr *et al.*, 2018) | 77 | 10/77 (13) | 77 | 20/77 (26) | Elagolix 600 mg qd (total 600 mg a day) vs Elagolix 600 mg qd + SD E2/NETA |
| *(Schlaff et al.*, 2020)  Elaris UF-1 | 104 | 7/104 (6.7) | 102 | 10/102 (9.8) | Elagolix 300 mg bd (total 600mg a day) vs placebo |
| *(Schlaff et al.*, 2020)  Elaris UF-1 | 104 | 7/104 (6.7) | 206 | 23/206 (11.2) | Elagolix 300 mg bd (total 600 mg a day) vs Elagolix 300 mg bd + SD E2/NETA |
| *(Schlaff et al.*, 2020)  Elaris UF-2 | 95 | 4/95 (4) | 94 | 9/94 (10) | Elagolix 300 mg bd (total 600mg a day) vs placebo |
| *(Schlaff et al.*, 2020)  Elaris UF-2 | 95 | 4/95 (4) | 189 | 14/189 (7.4) | Elagolix 300 mg bd (total 600 mg a day) vs Elagolix 300 mg bd + SD E2/NETA |
| *(Simon et al.*, 2020)  UF-EXTEND | 98 | 7/98 (7) | 218 | 23/218 (11) | Elagolix 300 mg bd (total 600 mg a day) vs Elagolix 300 mg bd + SD E2/NETA |

| **Outcome: Adverse Event (Fatigue)** | | | | | |
| --- | --- | --- | --- | --- | --- |
| **Author** | **Intervention group** | | **Control group** | | **Comments** |
|  | Number of participants analyzed | Percentage of participants  (%) | Number of participants analyzed | Percentage of participants  (%) |  |
| (Archer *et al.*, 2017) | 33 | 1/33 (3.0) | 16 | 0 | Cohort 4 Elagolix 100 mg bd (total 200 mg a day) vs Cohort 4 placebo |
| (Archer *et al.*, 2017) | 35 | 2/35 (5.7) | 18 | 1/18 (1.55) | Cohort 1 Elagolix 200 mg bd (total 400 mg a day) vs cohort 1 placebo |
| (Archer *et al.*, 2017) | 30 | 1/30 (3.3) | 16 | 0 | Cohort 2 Elagolix 300 mg bd (total 600 mg a day) vs cohort 2 placebo |
| (Archer *et al.*, 2017) | 32 | 4/32 (12.5) | 16 | 0 | Cohort 4 Elagolix 400 mg qd (total 400 mg a day) vs cohort 4 placebo |
| (Archer *et al.*, 2017) | 30 | 2/30 (6.7) | 16 | 0 | Elagolix 600 mg qd ( total 600 mg a day) vs placebo |
| (Archer *et al.*, 2017) | 35 | 2/35 (5.7) | 34 | 4/34 (11.8) | Elagolix 200 mg bd (total 400 mg a day) vs Elagolix 200 mg bd + LD E2/NETA |
| (Archer *et al.*, 2017) | 30 | 1/30 (3.3) | 27 | 1/27 (3.7) | Elagolix 300 mg bd (total 600 mg a day) vs Elagolix 300 mg bd + CEP |
| (Carr *et al.*, 2018) | 65 | 3/65 (4.6) | 65 | 2/65 (3.1) | Elagolix 300 mg bd (total 600 mg a day) vs placebo |
| (Carr *et al.*, 2018) | 65 | 3/65 (4.6) | 64 | 4/64 (6.3) | Elagolix 300 mg bd (total 600 mg a day) vs Elagolix 300 mg bd + LD E2/NETA |
| (Carr *et al.*, 2018) | 65 | 3/65 (4.6) | 65 | 3/65 (4.6) | Elagolix 300 mg bd (total 600 mg a day) vs Elagolix 300 mg bd + SD E2/NETA |
| (Carr *et al.*, 2018) | 77 | 0 | 78 | 3/78 (3.8) | Elagolix 600 mg qd (total 600 mg a day) vs placebo |
| (Carr *et al.*, 2018) | 77 | 0 | 76 | 1/76 (1.3) | Elagolix 600 mg qd (total 600 mg a day) vs Elagolix 600 mg qd + LD E2/NETA |
| (Carr *et al.*, 2018) | 77 | 0 | 77 | 5/77 (6.5) | Elagolix 600 mg qd (total 600 mg a day) vs Elagolix 600 mg qd + SD E2/NETA |
| *(Schlaff et al.*, 2020)  Elaris UF-1 | 104 | 1/104 (1.0) | 102 | 2/102 (2.0) | Elagolix 300 mg bd (total 600mg a day) vs placebo |
| *(Schlaff et al.*, 2020)  Elaris UF-1 | 104 | 1/104 (1.0) | 206 | 14/206 (6.8) | Elagolix 300 mg bd (total 600 mg a day) vs Elagolix 300 mg bd + SD E2/NETA |
| *(Schlaff et al.*, 2020)  Elaris UF-2 | 95 | 3/95 (3.0) | 94 | 5/94 (5) | Elagolix 300 mg bd (total 600mg a day) vs placebo |
| *(Schlaff et al.*, 2020)  Elaris UF-2 | 95 | 3/95 (3.0) | 189 | 10/189 (5.3) | Elagolix 300 mg bd (total 600 mg a day) vs Elagolix 300 mg bd + SD E2/NETA |
| *(Simon et al.*, 2020)  UF-EXTEND | 98 | 3/98 (3) | 218 | 16/218 (7) | Elagolix 300 mg bd (total 600 mg a day) vs Elagolix 300 mg bd + SD E2/NETA |

| **Outcome: Adverse Event (Hypertension)** | | | | | |
| --- | --- | --- | --- | --- | --- |
| **Author** | **Intervention group** | | **Control group** | | **Comments** |
|  | Number of participants analyzed | Percentage of participants  (%) | Number of participants analyzed | Percentage of participants  (%) |  |
| (Archer *et al.*, 2017) | 33 | 0 | 16 | 0 | Cohort 4 Elagolix 100 mg bd (total 200 mg a day) vs Cohort 4 placebo |
| (Archer *et al.*, 2017) | 35 | 0 | 18 | 1/18 (5.5) | Cohort 1 Elagolix 200 mg bd (total 400 mg a day) vs cohort 1 placebo |
| (Archer *et al.*, 2017) | 30 | 1/30 (3.3) | 16 | 0 | Cohort 2 Elagolix 300 mg bd (total 600 mg a day) vs cohort 2 placebo |
| (Archer *et al.*, 2017) | 32 | 0 | 16 | 0 | Cohort 4 Elagolix 400 mg qd (total 400 mg a day) vs cohort 4 placebo |
| (Archer *et al.*, 2017) | 30 | 3/30 (10) | 16 | 0 | Elagolix 600 mg qd ( total 600 mg a day) vs placebo |
| (Archer *et al.*, 2017) | 35 | 0 | 34 | 1/34 (2.9) | Elagolix 200 mg bd (total 400 mg a day) vs Elagolix 200 mg bd + LD E2/NETA |
| (Archer *et al.*, 2017) | 30 | 1/30 (3.3) | 27 | 0 | Elagolix 300 mg bd (total 600 mg a day) vs Elagolix 300 mg bd + CEP |
| (Carr *et al.*, 2018) | 65 | 0 | 65 | 0 | Elagolix 300 mg bd (total 600 mg a day) vs placebo |
| (Carr *et al.*, 2018) | 65 | 0 | 64 | 1/64 (1.5) | Elagolix 300 mg bd (total 600 mg a day) vs Elagolix 300 mg bd + LD E2/NETA |
| (Carr *et al.*, 2018) | 65 | 0 | 65 | 0 | Elagolix 300 mg bd (total 600 mg a day) vs Elagolix 300 mg bd + SD E2/NETA |
| (Carr *et al.*, 2018) | 77 | 0 | 78 | 0 | Elagolix 600 mg qd (total 600 mg a day) vs placebo |
| (Carr *et al.*, 2018) | 77 | 0 | 76 | 0 | Elagolix 600 mg qd (total 600 mg a day) vs Elagolix 600 mg qd + LD E2/NETA |
| (Carr *et al.*, 2018) | 77 | 0 | 77 | 0 | Elagolix 600 mg qd (total 600 mg a day) vs Elagolix 600 mg qd + SD E2/NETA |
| *(Simon et al.*, 2020)  UF-EXTEND | 98 | 4/98 (4) | 218 | 14/218 (6) | Elagolix 300 mg bd (total 600 mg a day) vs Elagolix 300 mg bd + SD E2/NETA |

**Continuous data**

| **Outcome: Reduction of monthly menstrual blood loss <80 ml** | | | | | | |
| --- | --- | --- | --- | --- | --- | --- |
| **Author** | **Unit of measurement** | **Intervention group** | | **Control group** | | **Comments** |
|  |  | Number of participants analyzed | Mean (SD) | Number of participants analyzed | Mean (SD) |  |
| *(Simon et al.*, 2020)  UF-EXTEND | ml | 59 | -151.7  95% CI of mean  (-202.2, -101.3) | 58 | -61.7 (-121.3, -2.1) | Elagolix 300 mg bd (total 600 mg a day) vs Elagolix 300 mg bd + SD E2/NETA |

| **Outcome: Reduction of >50% menstrual blood loss** | | | | | | |
| --- | --- | --- | --- | --- | --- | --- |
| **Author** | **Unit of measurement** | **Intervention group** | | **Control group** | | **Comments** |
|  |  | Number of participants analyzed | Mean (SD) | Number of participants analyzed | Mean (SD) |  |
| *(Simon et al.*, 2020)  UF-EXTEND | ml | 59 | -151.7  95% CI of mean  (-202.2, -101.3) | 58 | -61.7 (-121.3, -2.1) | Elagolix 300 mg bd (total 600 mg a day) vs Elagolix 300 mg bd + SD E2/NETA |

| **Outcome: Reduction in uterine volume** | | | | | | |
| --- | --- | --- | --- | --- | --- | --- |
| **Author** | **Unit of measurement** | **Intervention group** | | **Control group** | | **Comments** |
|  |  | Number of participants analyzed | Mean (SD) | Number of participants analyzed | Mean (SD) |  |
| (Archer *et al.*, 2017) | cm^3^ | 20 | -21.37 (24.84) | 9 | 18.72 (15.59) | Cohort 4 Elagolix 100 mg bd (total 200 mg a day) vs Cohort 4 placebo |
| (Archer *et al.*, 2017) | cm^3^ | 22 | -21.68 (29.80) | 12 | -8.62 (20.58) | Cohort 1 Elagolix 200 mg bd (total 400 mg a day) vs cohort 1 placebo |
| (Archer *et al.*, 2017) | cm^3^ | 20 | -33.25 (16.55) | 12 | -1.92 (17.52) | Cohort 2 Elagolix 300 mg bd (total 600 mg a day) vs cohort 2 placebo |
| (Archer *et al.*, 2017) | cm^3^ | 22 | -21.01 (26.79) | 9 | 18.72 (15.59) | Cohort 4 Elagolix 400 mg qd (total 400 mg a day) vs cohort 4 placebo |
| (Archer *et al.*, 2017) | cm^3^ | 20 | -27.99 (23.34) | 9 | 18.72 (15.59) | Elagolix 600 mg qd (total 600 mg a day) vs placebo |
| (Archer *et al.*, 2017) | cm^3^ | 22 | -21.68 (29.80) | 22 | -17.43 (19.51) | Elagolix 200 mg bd (total 400 mg a day) vs Elagolix 200 mg bd + LD E2/NETA |
| (Archer *et al.*, 2017) | cm^3^ | 20 | -33.25 (16.55) | 11 | -10.06 (30.93) | Elagolix 300 mg bd (total 600 mg a day) vs Elagolix 300 mg bd + CEP |
| (Carr *et al.*, 2018) | cm^3^ | 56 | -31.5 (31.44) | 58 | 15.9 (38.06) | Elagolix 300 mg bd (total 600 mg a day) vs placebo |
| (Carr *et al.*, 2018) | cm^3^ | 56 | -31.5 (31.44) | 56 | -22.0 (28.52) | Elagolix 300 mg bd (total 600 mg a day) vs Elagolix 300 mg bd + LD E2/NETA |
| (Carr *et al.*, 2018) | cm^3^ | 56 | -31.5 (31.44) | 56 | -11.8 (22.56) | Elagolix 300 mg bd (total 600 mg a day) vs Elagolix 300 mg bd + SD E2/NETA |
| (Carr *et al.*, 2018) | cm^3^ | 65 | -26.6 (28.26) | 72 | 11.6 (25.38) | Elagolix 600 mg qd (total 600 mg a day) vs placebo |
| (Carr *et al.*, 2018) | cm^3^ | 65 | -26.6 (28.26) | 58 | -11.5 (25.33) | Elagolix 600 mg qd (total 600 mg a day) vs Elagolix 600 mg qd + LD E2/NETA |
| (Carr *et al.*, 2018) | cm^3^ | 65 | -26.6 (28.26) | 64 | -6.7 (21.8) | Elagolix 600 mg qd (total 600 mg a day) vs Elagolix 600 mg qd + SD E2/NETA |
| (Schlaff *et al.*, 2020)  Elaris UF-1 | Percent change | 104 | -28.57 95% CI of mean ( -40.0, -25.71) | 102 | 8.57 (95% CI of mean (2.86, 17.14) | Elagolix 300 mg bd (total 600mg a day) vs placebo |
| (Schlaff *et al.*, 2020)  Elaris UF-1 | Percent change | 104 | -28.57 95% CI of mean ( -40.0, -25.71) | 206 | -5.71 95% CI of mean ( -11.43, 0) | Elagolix 300 mg bd (total 600 mg a day) vs Elagolix 300 mg bd + SD E2/NETA |
| (Schlaff *et al.*, 2020)  Elaris UF-2 | Percent change | 95 | -20.00 95% CI of mean ( -37.14, 0) | 94 | 10.00 95% CI of mean (0, 28.57) | Elagolix 300 mg bd (total 600mg a day) vs placebo |
| (Schlaff *et al.*, 2020)  Elaris UF-2 | Percent change | 95 | -20.00 95% CI of mean ( -37.14, 0) | 189 | 1.43 95% CI of mean (0, 11.42,) | Elagolix 300 mg bd (total 600 mg a day) vs Elagolix 300 mg bd + SD E2/NETA |
| *(Simon et al.*, 2020)  UF-EXTEND | cm^3^ | 80 | -200.0  95% CI of mean  (-250.0, -160.0) | 177 | -66.6  95% CI of mean  (-150.0, -20.0) | Elagolix 300 mg bd (total 600 mg a day) vs Elagolix 300 mg bd + SD E2/NETA |
| **Outcome: Reduction in fibroid volume** | | | | | | |
| **Author** | **Unit of measurement** | **Intervention group** | | **Control group** | | **Comments** |
|  |  | Number of participants analyzed | Mean (SD) | Number of participants analyzed | Mean (SD) |  |
| (Archer *et al.*, 2017) | cm^3^ | 18 | -22.19 (51.14) | 9 | -7.26 (36.35) | Cohort 4 Elagolix 100 mg bd (total 200 mg a day) vs Cohort 4 placebo |
| (Archer *et al.*, 2017) | cm^3^ | 22 | -38.52 (41.72) | 12 | -2.05 (71.83) | Cohort 1 Elagolix 200 mg bd (total 400 mg a day) vs cohort 1 placebo |
| (Archer *et al.*, 2017) | cm^3^ | 20 | -35.79 (24.49) | 12 | 6.70 (45.42) | Cohort 2 Elagolix 300 mg bd (total 600 mg a day) vs cohort 2 placebo |
| (Archer *et al.*, 2017) | cm^3^ | 19 | 14.23 (187.83) | 9 | -7.26 (36.35) | Cohort 4 Elagolix 400 mg qd (total 400 mg a day) vs cohort 4 placebo |
| (Archer *et al.*, 2017) | cm^3^ | 18 | -16.60 (39.61) | 9 | -7.26 (36.35) | Elagolix 600 mg qd (total 600 mg a day) vs placebo |
| (Archer *et al.*, 2017) | cm^3^ | 22 | -38.52 (41.72) | 22 | -25.77 (46.64) | Elagolix 200 mg bd (total 400 mg a day) vs Elagolix 200 mg bd + LD E2/NETA |
| (Archer *et al.*, 2017) | cm^3^ | 20 | -35.79 (24.49) | 10 | -4.94 (100.68) | Elagolix 300 mg bd (total 600 mg a day) vs Elagolix 300 mg bd + CEP |
| (Carr *et al.*, 2018) | cm^3^ | 53 | -39.6 (28.66) | 53 | 4.6 (48.59) | Elagolix 300 mg bd (total 600 mg a day) vs placebo |
| (Carr *et al.*, 2018) | cm^3^ | 53 | -39.6 (28.66) | 54 | -24.0 (29.93) | Elagolix 300 mg bd (total 600 mg a day) vs Elagolix 300 mg bd + LD E2/NETA |
| (Carr *et al.*, 2018) | cm^3^ | 53 | -39.6 (28.66) | 51 | -12.9 (46.2) | Elagolix 300 mg bd (total 600 mg a day) vs Elagolix 300 mg bd + SD E2/NETA |
| (Carr *et al.*, 2018) | cm^3^ | 59 | -36.4 (30.07) | 66 | 0.1 (28.82) | Elagolix 600 mg qd (total 600 mg a day) vs placebo |
| (Carr *et al.*, 2018) | cm^3^ | 59 | -36.4 (30.07) | 55 | -16.6 (32.65) | Elagolix 600 mg qd (total 600 mg a day) vs Elagolix 600 mg qd + LD E2/NETA |
| (Carr *et al.*, 2018) | cm^3^ | 59 | -36.4 (30.07) | 60 | -1.6 (42.75) | Elagolix 600 mg qd (total 600 mg a day) vs Elagolix 600 mg qd + SD E2/NETA |
| (Schlaff *et al.*, 2020)  Elaris UF-1 | Percent change | 102 | -28.57 95% CI of mean (-42.85, -14.28) | 100 | 14.29 95% CI of mean (2.85, 28.57) | Elagolix 300 mg bd (total 600mg a day) vs placebo |
| (Schlaff *et al.*, 2020)  Elaris UF-1 | Percent change | 102 | -28.57  95% CI of mean (-42.85, -14.28) | 203 | -5.71 95% CI of mean (-14.28, 2.85) | Elagolix 300 mg bd (total 600 mg a day) vs Elagolix 300 mg bd + SD E2/NETA |
| (Schlaff *et al.*, 2020)  Elaris UF-2 | Percent change | 95 | -20.00 95% CI of mean (-31.42, 0) | 92 | -2.86 95% CI of mean (-12.85, 0) | Elagolix 300 mg bd (total 600mg a day) vs placebo |
| (Schlaff *et al.*, 2020)  Elaris UF-2 | Percent change | 95 | -20.00 95% CI of mean (-31.42, 0) | 186 | 0  95% CI of mean (-8.57, 0) | Elagolix 300 mg bd (total 600 mg a day) vs Elagolix 300 mg bd + SD E2/NETA |
| *(Simon et al.*, 2020)  UF-EXTEND | cm^3^ | 77 | -26.6  95% CI of mean (-48.8, -8.8) | 169 | 2.0  95% CI of mean (-6.67, 8.89) | Elagolix 300 mg bd (total 600 mg a day) vs Elagolix 300 mg bd + SD E2/NETA |

| **Outcome: Symptom severity (UFS-QoL)** | | | | | | |
| --- | --- | --- | --- | --- | --- | --- |
| **Author** | **Unit of measurement** | **Intervention group** | | **Control group** | | **Comments** |
|  |  | Number of participants analyzed | Mean (SD) | Number of participants analyzed | Mean (SD) |  |
| (Archer *et al.*, 2017) | Unit on a scale | 21 | -33.2 (28.17) | 9 | -19.6 (32.80) | Cohort 4 Elagolix 100 mg bd (total 200 mg a day) vs Cohort 4 placebo |
| (Archer *et al.*, 2017) | Unit on a scale | 26 | -31.6 (28.87) | 13 | -21.4 (20.63) | Cohort 1 Elagolix 200 mg bd (total 400 mg a day) vs cohort 1 placebo |
| (Archer *et al.*, 2017) | Unit on a scale | 25 | -44.1 (22.12) | 13 | -12.0 (22.49) | Cohort 2 Elagolix 300 mg bd (total 600 mg a day) vs cohort 2 placebo |
| (Archer *et al.*, 2017) | Unit on a scale | 23 | -39.0 (24.70) | 9 | -19.6 (32.80) | Cohort 4 Elagolix 400 mg qd (total 400 mg a day) vs cohort 4 placebo |
| (Archer *et al.*, 2017) | Unit on a scale | 20 | -36.4 (24.74) | 9 | -19.6 (32.80) | Elagolix 600 mg qd (total 600 mg a day) vs placebo |
| (Archer *et al.*, 2017) | Unit on a scale | 26 | -31.6 (28.87) | 22 | -20.3 (25.35) | Elagolix 200 mg bd (total 400 mg a day) vs Elagolix 200 mg bd + LD E2/NETA |
| (Archer *et al.*, 2017) | Unit on a scale | 25 | -44.1 (22.12) | 14 | -39.1 (24.09) | Elagolix 300 mg bd (total 600 mg a day) vs Elagolix 300 mg bd + CEP |
| (Carr et al., 2018) | Unit on a scale | 62 | -45.6 (2.7) | 64 | -17.9 (2.6) | Elagolix 300 mg bd (total 600 mg a day) vs placebo |
| (Carr et al., 2018) | Unit on a scale | 62 | -45.6 (2.7) | 61 | -38.8 (2.7) | Elagolix 300 mg bd (total 600 mg a day) vs Elagolix 300 mg bd + LD E2/NETA |
| (Carr et al., 2018) | Unit on a scale | 62 | -45.6 (2.7) | 62 | -36.5 (2.6) | Elagolix 300 mg bd (total 600 mg a day) vs Elagolix 300 mg bd + SD E2/NETA |
| (Carr et al., 2018) | Unit on a scale | 71 | -44.2 (2.7) | 76 | -21.9 (2.6) | Elagolix 600 mg qd (total 600 mg a day) vs placebo |
| (Carr et al., 2018) | Unit on a scale | 71 | -44.2 (2.7) | 73 | -36.2 (2.7) | Elagolix 600 mg qd (total 600 mg a day) vs Elagolix 600 mg qd + LD E2/NETA |
| (Carr et al., 2018) | Unit on a scale | 71 | -44.2 (2.7) | 76 | -32.6 (2.6) | Elagolix 600 mg qd (total 600 mg a day) vs Elagolix 600 mg qd + SD E2/NETA |
| (Diamond et al., 2017) | Unit on a scale | 49 | 64.7 (-49.8) | 49 | 62.8 (-19.2) | Elagolix 300 mg bd (total 600 mg a day) vs placebo |
| (Diamond et al., 2017) | Unit on a scale | 49 | 64.7 (-49.8) | 52 | 61.8 (-40.7) | Elagolix 300 mg bd (total 600 mg a day) vs Elagolix 300 mg bd + LD E2/NETA |
| (Diamond et al., 2017) | Unit on a scale | 49 | 64.7 (-49.8) | 51 | 63.5 (-37.2) | Elagolix 300 mg bd (total 600 mg a day) vs Elagolix 300 mg bd + SD E2/NETA |
| (Schlaff et al., 2020)  ELARIS UF-1 | Unit on a scale | 103 | -49  95% CI of mean (-51.0, -46) | 102 | -10.3 ± 2.25  Mean ± SE | Elagolix 300 mg bd (total 600 mg a day) vs placebo |
| (Schlaff et al., 2020)  ELARIS UF-1 | Unit on a scale | 103 | -49  95% CI of mean (-51.0, -46) | 206 | -33.2 ± 6.1  Mean ± SE | Elagolix 300 mg bd (total 600 mg a day) vs Elagolix 300 mg bd + SD E2/NETA |
| (Schlaff et al., 2020)  ELARIS UF-2 | Unit on a scale | 94 | -50  95% CI of mean (-53.0, -48) | 92 | -7.9 ± 2.28  Mean ± SE | Elagolix 300 mg bd (total 600 mg a day) vs placebo |
| (Schlaff et al., 2020)  ELARIS UF-2 | Unit on a scale | 94 | -50  95% CI of mean (-53.0, -48) | 186 | -41.4 ± 6.0  Mean ± SE | Elagolix 300 mg bd (total 600 mg a day) vs Elagolix 300 mg bd + SD E2/NETA |
| *(Simon et al.*, 2020)  UF-EXTEND | Unit on scalen | 73 | -54.0  95% CI of mean (-62.0, -52.0) | 173 | -40.0  95% CI of mean (-44.0, -36.0)) | Elagolix 300 mg bd (total 600 mg a day) vs Elagolix 300 mg bd + SD E2/NETA |

| **Outcome: Health-related quality of life (UFS-QoL)** | | | | | | |
| --- | --- | --- | --- | --- | --- | --- |
| **Author** | **Unit of measurement** | **Intervention group** | | **Control group** | | **Comments** |
|  |  | Number of participants analyzed | Mean (SD) | Number of participants analyzed | Mean (SD) |  |
| (Archer *et al.*, 2017) | Unit on a scale | 21 | 29.1 (30.96) | 9 | 16.3 (30.43) | Cohort 4 Elagolix 100 mg bd (total 200 mg a day) vs Cohort 4 placebo |
| (Archer *et al.*, 2017) | Unit on a scale | 25 | 36.0 (27.91) | 13 | 18.3 (36.66) | Cohort 1 Elagolix 200 mg bd (total 400 mg a day) vs cohort 1 placebo |
| (Archer *et al.*, 2017) | Unit on a scale | 25 | 33.5 (29.42) | 13 | 11.0 (29.90) | Cohort 2 Elagolix 300 mg bd (total 600 mg a day) vs cohort 2 placebo |
| (Archer *et al.*, 2017) | Unit on a scale | 22 | 35.3 (21.87) | 9 | 16.3 (30.43) | Cohort 4 Elagolix 400 mg qd (total 400 mg a day) vs cohort 4 placebo |
| (Archer *et al.*, 2017) | Unit on a scale | 20 | 29.9 (30.72) | 9 | 16.3 (30.43) | Elagolix 600 mg qd (total 600 mg a day) vs placebo |
| (Archer *et al.*, 2017) | Unit on a scale | 25 | 36.0 (27.91) | 22 | 28.6 (24.12) | Elagolix 200 mg bd (total 400 mg a day) vs Elagolix 200 mg bd + LD E2/NETA |
| (Archer *et al.*, 2017) | Unit on a scale | 25 | 33.5 (29.42) | 14 | 33.1 (30.34) | Elagolix 300 mg bd (total 600 mg a day) vs Elagolix 300 mg bd + CEP |
| (Carr et al., 2018) | Unit on a scale | 62 | 44.9 (2.9) | 64 | 14.7 (2.8) | Elagolix 300 mg bd (total 600 mg a day) vs placebo |
| (Carr et al., 2018) | Unit on a scale | 62 | 44.9 (2.9) | 61 | 41.2 (2.9) | Elagolix 300 mg bd (total 600 mg a day) vs Elagolix 300 mg bd + LD E2/NETA |
| (Carr et al., 2018) | Unit on a scale | 62 | 44.9 (2.9) | 62 | 36.0 (2.9) | Elagolix 300 mg bd (total 600 mg a day) vs Elagolix 300 mg bd + SD E2/NETA |
| (Carr et al., 2018) | Unit on a scale | 71 | 43.5 (3.1) | 76 | 24.8 (3.0) | Elagolix 600 mg qd (total 600 mg a day) vs placebo |
| (Carr et al., 2018) | Unit on a scale | 71 | 43.5 (3.1) | 73 | 36.3 (3.1) | Elagolix 600 mg qd (total 600 mg a day) vs Elagolix 600 mg qd + LD E2/NETA |
| (Carr et al., 2018) | Unit on a scale | 71 | 43.5 (3.1) | 76 | 34.7 (3.0) | Elagolix 600 mg qd (total 600 mg a day) vs Elagolix 600 mg qd + SD E2/NETA |
| (Diamond et al., 2017) | Unit on a scale | 49 | 39.3 (46.9) | 49 | 37.7 (16.0) | Elagolix 300 mg bd (total 600 mg a day) vs placebo |
| (Diamond et al., 2017) | Unit on a scale | 49 | 39.3 (46.9) | 52 | 41.1 (42.4) | Elagolix 300 mg bd (total 600 mg a day) vs Elagolix 300 mg bd + LD E2/NETA |
| (Diamond et al., 2017) | Unit on a scale | 49 | 39.3 (46.9) | 51 | 37.7 (38.6) | Elagolix 300 mg bd (total 600 mg a day) vs Elagolix 300 mg bd + SD E2/NETA |
| (Schlaff et al., 2020)  ELARIS UF-1 | Unit on a scale | 103 | 48  95% CI of mean (45, 50) | 102 | 10.9 ± 2.27  Mean ± SE | Elagolix 300 mg bd (total 600 mg a day) vs placebo |
| (Schlaff et al., 2020)  ELARIS UF-1 | Unit on a scale | 103 | 48  95% CI of mean (45, 50 | 206 | 38.0 ± 1.62  Mean ± SE | Elagolix 300 mg bd (total 600 mg a day) vs Elagolix 300 mg bd + SD E2/NETA |
| (Schlaff et al., 2020)  ELARIS UF-2 | Unit on a scale | 94 | 48  95% CI of mean (45, 50) | 92 | 6.5 ± 2.32  Mean ± SE | Elagolix 300 mg bd (total 600 mg a day) vs placebo |
| (Schlaff et al., 2020)  ELARIS UF-2 | Unit on a scale | 94 | 48  95% CI of mean (45, 50) | 186 | 42 ± 1.62  Mean ± SE | Elagolix 300 mg bd (total 600 mg a day) vs Elagolix 300 mg bd + SD E2/NETA |
| *(Simon et al.*, 2020)  UF-EXTEND | Unit on scalen | 73 | 52.8  95% CI of mean (46.0, 58.0) | 173 | 40.0  95% CI of mean (36.0, 46.0) | Elagolix 300 mg bd (total 600 mg a day) vs Elagolix 300 mg bd + SD E2/NETA |

* not reported in the article

| **Outcome: Haemoglobin level** | | | | | | |
| --- | --- | --- | --- | --- | --- | --- |
| **Author** | **Unit of measurement** | **Intervention group** | | **Control group** | | **Comments** |
|  |  | Number of participants analyzed | Mean (SD) | Number of participants analyzed | Mean (SD) |  |
| (Archer *et al.*, 2017) | g/dl | 24 | 1.30 (1.19) | 11 | -0.43 (1.28) | Cohort 4 Elagolix 100 mg bd (total 200 mg a day) vs Cohort 4 placebo |
| (Archer *et al.*, 2017) | g/dl | 27 | 1.13 (1.29) | 14 | 0.28 (1.33) | Cohort 1 Elagolix 200 mg bd (total 400 mg a day) vs cohort 1 placebo |
| (Archer *et al.*, 2017) | g/dl | 25 | 1.19 (0.85) | 14 | 0.31 (1.20) | Cohort 2 Elagolix 300 mg bd (total 600 mg a day) vs cohort 2 placebo |
| (Archer *et al.*, 2017) | g/dl | 23 | 1.18 (0.99) | 11 | -0.43 (1.28) | Cohort 4 Elagolix 400 mg qd (total 400 mg a day) vs cohort 4 placebo |
| (Archer *et al.*, 2017) | g/dl | 23 | 1.40 (1.18) | 11 | -0.43 (1.28) | Elagolix 600 mg qd (total 600 mg a day) vs placebo |
| (Archer *et al.*, 2017) | g/dl | 27 | 1.13 (1.29) | 28 | 0.92 (0.81) | Elagolix 200 mg bd (total 400 mg a day) vs Elagolix 200 mg bd + LD E2/NETA |
| (Archer *et al.*, 2017) | g/dl | 25 | 1.19 (0.85) | 21 | 1.54 (1.81) | Elagolix 300 mg bd (total 600 mg a day) vs Elagolix 300 mg bd + CEP |
| (Carr et al., 2018) | g/dl | 61 | 1.9 (0.17) | 64 | 0.6 (0.17) | Elagolix 300 mg bd (total 600 mg a day) vs placebo |
| (Carr et al., 2018) | g/dl | 61 | 1.9 (0.17) | 59 | 1.9 (0.17) | Elagolix 300 mg bd (total 600 mg a day) vs Elagolix 300 mg bd + LD E2/NETA |
| (Carr et al., 2018) | g/dl | 61 | 1.9 (0.17) | 61 | 1.4 (0.17) | Elagolix 300 mg bd (total 600 mg a day) vs Elagolix 300 mg bd + SD E2/NETA |
| (Carr et al., 2018) | g/dl | 71 | 1.4 (0.16) | 76 | 0.3 (0.15) | Elagolix 600 mg qd (total 600 mg a day) vs placebo |
| (Carr et al., 2018) | g/dl | 71 | 1.4 (0.16) | 72 | 1.1 (0.16) | Elagolix 600 mg qd (total 600 mg a day) vs Elagolix 600 mg qd + LD E2/NETA |
| (Carr et al., 2018) | g/dl | 71 | 1.4 (0.16) | 74 | 1.2 (0.16) | Elagolix 600 mg qd (total 600 mg a day) vs Elagolix 600 mg qd + SD E2/NETA |
| *(Simon et al.*, 2020)  UF-EXTEND | g/dl | 59 | 70.6  95% CI of mean (48.9, 92.3) | 58 | 36.4  95% CI of mean (7.9, 64.8) | Elagolix 300 mg bd (total 600 mg a day) vs Elagolix 300 mg bd + SD E2/NETA |

| **Outcome: Bone mineral density (lumbar spine)** | | | | | | |
| --- | --- | --- | --- | --- | --- | --- |
| **Author** | **Unit of measurement** | **Intervention group** | | **Control group** | | **Comments** |
|  |  | Number of participants analyzed | Mean (SD) | Number of participants analyzed | Mean (SD) |  |
| (Carr et al., 2018) | g/cm² | 65 | -3.8  95% CI of mean (-4.26, -2.78) | 65 | 0.9  95% CI of mean (0.08, 1.65) | Elagolix 300 mg bd (total 600 mg a day) vs placebo |
| (Carr et al., 2018) | g/cm² | 65 | -3.8  95% CI of mean (-4.26, -2.78) | 64 | -1.6  95% CI of mean (-2.26, -0.78) | Elagolix 300 mg bd (total 600 mg a day) vs Elagolix 300 mg bd + LD E2/NETA |
| (Carr et al., 2018) | g/cm² | 65 | -3.8  95% CI of mean (-4.26, -2.78) | 65 | -0.1  95% CI of mean (-0.87, 0.6) | Elagolix 300 mg bd (total 600 mg a day) vs Elagolix 300 mg bd + SD E2/NETA |
| (Carr et al., 2018) | g/cm² | 77 | -3.4  95% CI of mean (-4.0, -2.63) | 78 | -0.1  95% CI of mean (-0.9, 0.54) | Elagolix 600 mg qd (total 600 mg a day) vs placebo |
| (Carr et al., 2018) | g/cm² | 77 | -3.4  95% CI of mean (-4.0, -2.63) | 76 | -1.2  95% CI of mean (- 2.0, 0.49) | Elagolix 600 mg qd (total 600 mg a day) vs Elagolix 600 mg qd + LD E2/NETA |
| (Carr et al., 2018) | g/cm² | 77 | -3.4  95% CI of mean (-4.0, -2.63) | 77 | -1.1  95% CI of mean (-1.8, -0.36) | Elagolix 600 mg qd (total 600 mg a day) vs Elagolix 600 mg qd + SD E2/NETA |
| (Schlaff et al., 2020)  ELARIS UF-1 | g/cm² | 74 | -2.95  95% CI of mean (-3.49 to -2.01) | 82 | -0.21  95% CI of mean (-0.5, 0.05) | Elagolix 300 mg bd (total 600 mg a day) vs placebo |
| (Schlaff et al., 2020)  ELARIS UF-1 | g/cm² | 74 | -2.95  95% CI of mean (-3.49 to -2.01) | 159 | -0.76  95% CI of mean (-1.18 to 0.08) | Elagolix 300 mg bd (total 600 mg a day) vs Elagolix 300 mg bd + SD E2/NETA |
| (Schlaff et al., 2020)  ELARIS UF-2 | g/cm² | 65 | -2.94  95% CI of mean (-3.80 to -1.96) | 68 | -0.06  95% CI of mean (-0.38, 0.27) | Elagolix 300 mg bd (total 600 mg a day) vs placebo |
| (Schlaff et al., 2020)  ELARIS UF-2 | g/cm² | 65 | -2.94  95% CI of mean (-3.80 to -1.96) | 147 | -0.61  95% CI of mean (-1.33 to 0.23) | Elagolix 300 mg bd (total 600 mg a day) vs Elagolix 300 mg bd + SD E2/NETA |
| *(Simon et al.*, 2020)  UF-EXTEND | g/cm² | 81 | -4.8  95% CI of mean (-5.4, -4.1)) | 176 | -1.5  95% CI of mean (-1.8, -1.0) | Elagolix 300 mg bd (total 600 mg a day) vs Elagolix 300 mg bd + SD E2/NETA |

| **Outcome: Bone mineral density (total hip)** | | | | | | |
| --- | --- | --- | --- | --- | --- | --- |
| **Author** | **Unit of measurement** | **Intervention group** | | **Control group** | | **Comments** |
|  |  | Number of participants analyzed | Mean (SD) | Number of participants analyzed | Mean (SD) |  |
| (Carr et al., 2018) | g/cm² | 65 | -2.2  95% CI of mean (--2.72, -1.63) | 65 | 0.8  95% CI of mean (-0.18, 1.36) | Elagolix 300 mg bd (total 600 mg a day) vs placebo |
| (Carr et al., 2018) | g/cm² | 65 | -2.2  95% CI of mean (--2.72, -1.63) | 64 | -1.1  95% CI of mean (-1.63, -0.45) | Elagolix 300 mg bd (total 600 mg a day) vs Elagolix 300 mg bd + LD E2/NETA |
| (Carr et al., 2018) | g/cm² | 65 | -2.2  95% CI of mean (--2.72, -1.63) | 65 | -0.6  95% CI of mean (-1.27,0) | Elagolix 300 mg bd (total 600 mg a day) vs Elagolix 300 mg bd + SD E2/NETA |
| (Carr et al., 2018) | g/cm² | 77 | -1.8  95% CI of mean (-2.27, 1.27) | 78 | 0.1  95% CI of mean (-0.4, 0.58) | Elagolix 600 mg qd (total 600 mg a day) vs placebo |
| (Carr et al., 2018) | g/cm² | 77 | -1.8  95% CI of mean (-2.27, 1.27) | 76 | -0.9  95% CI of mean (-1.45, -0.36) | Elagolix 600 mg qd (total 600 mg a day) vs Elagolix 600 mg qd + LD E2/NETA |
| (Carr et al., 2018) | g/cm² | 77 | -1.8  95% CI of mean (-2.27, 1.27) | 77 | -0.2  95% CI of mean (-0.72, 0.36) | Elagolix 600 mg qd (total 600 mg a day) vs Elagolix 600 mg qd + SD E2/NETA |
| (Schlaff et al., 2020)  ELARIS UF-1 | g/cm² | 74 | -2.12  95% CI of mean (-2.39 to -1.14) | 82 | -0.35  95% CI of mean (-0.55, -0.11) | Elagolix 300 mg bd (total 600 mg a day) vs placebo |
| (Schlaff et al., 2020)  ELARIS UF-1 | g/cm² | 74 | -2.12  95% CI of mean (-2.39 to -1.14) | 159 | -0.15  95% CI of mean (-0.33 to 0.73) | Elagolix 300 mg bd (total 600 mg a day) vs Elagolix 300 mg bd + SD E2/NETA |
| (Schlaff et al., 2020)  ELARIS UF-2 | g/cm² | 65 | -1.80  95% CI of mean (-2.48 to -1.17) | 68 | 0.03  95% CI of mean (- 0.22, 0.27) | Elagolix 300 mg bd (total 600 mg a day) vs placebo |
| (Schlaff et al., 2020)  ELARIS UF-2 | g/cm² | 65 | -1.80  95% CI of mean (-2.48 to -1.17) | 147 | -0.12  95% CI of mean (-2.24 to -1.11) | Elagolix 300 mg bd (total 600 mg a day) vs Elagolix 300 mg bd + SD E2/NETA |
| *(Simon et al.*, 2020)  UF-EXTEND | g/cm² | 81 | -3.4  95% CI of mean (-3.85, -2.75) | 176 | -0.6  95% CI of mean (-1.25, -0.38) | Elagolix 300 mg bd (total 600 mg a day) vs Elagolix 300 mg bd + SD E2/NETA |

| **Outcome: Bone mineral density (femoral neck)** | | | | | | |
| --- | --- | --- | --- | --- | --- | --- |
| **Author** | **Unit of measurement** | **Intervention group** | | **Control group** | | **Comments** |
|  |  | Number of participants analyzed | Mean (SD) | Number of participants analyzed | Mean (SD) |  |
| (Carr et al., 2018) | g/cm² | 65 | -2.8  95% CI of mean (-3.76, -1.77) | 65 | 0.5  95% CI of mean (-0.61, 1.46) | Elagolix 300 mg bd (total 600 mg a day) vs placebo |
| (Carr et al., 2018) | g/cm² | 65 | -2.8  95% CI of mean (-3.76, -1,77) | 64 | -1.7  95% CI of mean (-2.77, 0.77) | Elagolix 300 mg bd (total 600 mg a day) vs Elagolix 300 mg bd + LD E2/NETA |
| (Carr et al., 2018) | g/cm² | 65 | -2.8  95% CI of mean (-3.76, -1,77) | 65 | -0.5  95% CI of mean (-1.54, o.46) | Elagolix 300 mg bd (total 600 mg a day) vs Elagolix 300 mg bd + SD E2/NETA |
| (Carr et al., 2018) | g/cm² | 77 | -1.8  95% CI of mean (-2.77, 0.92) | 78 | -0.2  95% CI of mean (-1.23, 0.69) | Elagolix 600 mg qd (total 600 mg a day) vs placebo |
| (Carr et al., 2018) | g/cm² | 77 | -1.8  95% CI of mean (-2.77, 0.92) | 76 | -1.2  95% CI of mean (-2.15, -0.15) | Elagolix 600 mg qd (total 600 mg a day) vs Elagolix 600 mg qd + LD E2/NETA |
| (Carr et al., 2018) | g/cm² | 77 | -1.8  95% CI of mean (-2.77, 0.92) | 77 | -0.5  95% CI of mean (-1.53, 0.38) | Elagolix 600 mg qd (total 600 mg a day) vs Elagolix 600 mg qd + SD E2/NETA |
| (Schlaff et al., 2020)  ELARIS UF-1 | g/cm² | 74 | -2.46  95% CI of mean (-3.17 to -1.04) | 82 | -0.35  95% CI of mean (-0.77, 0.05) | Elagolix 300 mg bd (total 600 mg a day) vs placebo |
| (Schlaff et al., 2020)  ELARIS UF-1 | g/cm² | 74 | -2.46  95% CI of mean (-3.17 to -1.04) | 159 | -0.83  95% CI of mean (-1.39 to 0.43) | Elagolix 300 mg bd (total 600 mg a day) vs Elagolix 300 mg bd + SD E2/NETA |
| (Schlaff et al., 2020)  ELARIS UF-2 | g/cm² | 65 | -1.19  95% CI of mean (-2.26 to 0.09) | 68 | -0.10  95% CI of mean (-0.55, 0.33) | Elagolix 300 mg bd (total 600 mg a day) vs placebo |
| (Schlaff et al., 2020)  ELARIS UF-2 | g/cm² | 65 | -1.19  95% CI of mean (-2.26 to 0.09) | 147 | -0.39  95% CI of mean (-1.29 to 0.71) | Elagolix 300 mg bd (total 600 mg a day) vs Elagolix 300 mg bd + SD E2/NETA |
| *(Simon et al.*, 2020)  UF-EXTEND | g/cm² | 81 | -3.0  95% CI of mean (-3.75, -2.25) | 176 | -1.0  95% CI of mean (-1.63, 0.38) | Elagolix 300 mg bd (total 600 mg a day) vs Elagolix 300 mg bd + SD E2/NETA |
